# Disparities in adolescent mental health-related National Health Service presentations in England: a secondary school cohort study

**DOI:** 10.1136/bmjph-2025-004281

**Published:** 2026-10-06

**Authors:** Kate M Lewis, Helen Dale, Dan Lewer, Rocío Méndez Pineda, Johnny Downs, Ruth M Blackburn

**Affiliations:** 1 Great Ormond Street Institute of Child Health, UCL, London, UK; 2 Population Health Improvement UK (PHI-UK), National Research Network, UK; 3 Bradford Teaching Hospitals NHS Foundation Trust, Bradford Institute for Health Research, Bradford, UK; 4 Department of Child and Adolescent Psychiatry, King’s College London, London, UK

**Keywords:** Adolescent, Mental Health, Health Services Accessibility, Sociodemographic Factors

## Abstract

**Introduction:**

Evidence on demographic and socioeconomic differences in adolescent mental health service use *across multiple healthcare settings* is vital for equitable health policy and service responses. We quantified inequalities in referrals to National Health Service (NHS)-funded mental health services and NHS hospital use for mental health concerns by social strata for secondary school pupils in England.

**Methods:**

We used linked education-health records (ECHILD) to create a cohort of pupils entering state-funded secondary schools in England (age 11 years/year 7) from 2012/13 to 2021/22. We measured rates of service use by gender, racial-ethnic group, region of residence, free school meal eligibility and area-level deprivation. Contacts were captured in the Mental Health Services Data Set and Hospital Episode Statistics. Pupils were followed up until service contact, age 21 years, death or study end (31 August 2022).

**Results:**

Of 5 629 720 pupils, 980 648 (17.4%) were referred to NHS-funded mental health services; a rate of 45.2 per 1000 person-years (95% CI 45.1 to 45.2). Referral rates were higher in females (55.5, 95% CI 55.4 to 55.7) than males (35.7, 95% CI 35.6 to 35.8) and were highest in the North East (58.2, 95% CI 57.7 to 58.7). Rates were highest among White Irish travellers (70.8, 95% CI 66.2 to 75.8) and lowest among Pakistani (20.8, 95% CI 20.5 to 21.1) pupils. Pupils eligible for free school meals had particularly high rates (75.3, 95% CI 75.0 to 75.6), and rates increased with quintiles of area-level deprivation. Sociodemographic patterns were consistent across referrals, emergency department attendances, and hospital admissions.

**Conclusions:**

We find marked variation in mental health service use across social strata but consistency across different service types, likely reflecting differences in underlying need, symptom recognition, help-seeking, referral pathways, recording practice and the availability and acceptability of services.

**WHAT IS ALREADY KNOWN ON THIS TOPIC**

- Improving access to mental health services for young people is a priority for the English National Health Service, but there is limited integrated population-level information across education and health systems about rates of contacts across multiple healthcare services and with comprehensive coverage of England.

**WHAT THIS STUDY ADDS**

- Almost one fifth of pupils were referred to mental health services, with stark variation by gender, racial-ethnic group, free school meal eligibility, neighbourhood deprivation and region of residence.
- Relative patterns of contacts by social strata were similar across emergency department attendances and hospital admissions.

**HOW THIS STUDY MIGHT AFFECT RESEARCH, PRACTICE OR POLICY**

- Clear differences in service contacts across social strata likely reflect a combination of differences in underlying need, symptom recognition, help-seeking, referral pathways, recording practices, and the availability and acceptability of NHS services across population strata.
- We find no evidence of disproportionate use of a single service type after accounting for age and follow-up year, suggesting that policies addressing inequities should span across all services and must seek to address social determinants of health, including differential barriers to service use.

## Introduction

 Adolescence is a key time for the emergence of mental health conditions. A meta-analysis in 2022 found that half of all mental disorders worldwide begin before the age of 18 years.^1^ While the benefits of interventions are thought to be greatest when implemented at the point of onset, considerable unmet need in young people is reported.^1,2^ In England, an estimated three-fifths of all under 18 year olds with a diagnosable mental health problem did not access the National Health Service (NHS) for mental health in 2023/24, despite such services being free for residents to access at the point of use.^3^ There are recognised inequities, defined here as avoidable differences in health that are unfair and unjust,^4^ in the prevalence of mental health disorders, driven by the unequal distribution of the social determinants of health and, subsequently, differential exposure to social, economic and environmental stressors.^5^ For example, 55% of 11–16 year olds in families in England with self-reported financial worries had a probable mental disorder compared with 27% without these worries.^6^ Access to formal mental health services is also likely to be inequitable.^7^ Qualitative research has highlighted barriers in both the accessibility and acceptability of services (including information provision, service capacity, and the cultural and gender-related fit of professional workforces) and in the ability of populations to access services (including health beliefs, stigma or shame, and practical difficulties such as transport).^8–12^

Improving access for mental health services and narrowing inequalities for 0–17-year-olds for ‘certain ethnic groups, age, gender and deprivation’ are key NHS priorities.^13,14^ Despite these policy priorities, detailed population-based information on mental health-related contacts across multiple healthcare settings and social strata, including region of residence, is lacking. Establishing national patterns of service contacts is a critical step to ensuring equitable service provision, particularly given rising demand for services, including admissions to acute paediatric wards for mental health concerns.^6,15^ Estimating disparities in contacts across services may highlight where inequities exist and for whom. For example, adolescents who face barriers to accessing outpatient mental health support may disproportionately end up in emergency care. Using nationally linked education and health records in England, this study describes rates of mental health-related NHS presentations among adolescents enrolled in state-funded secondary schools. We address three descriptive research questions: (1) what are the rates of mental health service referrals, emergency department (ED) attendances and emergency hospital admissions among pupils in England; (2) how do these rates vary across key social strata (gender, region of residence, racial-ethnic group, socioeconomic deprivation, free school meal eligibility) and other high-risk population groups (care-experienced pupils, those with chronic conditions, those with special educational needs and disability (SEND) provision and those persistently absent from education); and (3) do patterns of service contact appear consistent across the three service types? Throughout, these descriptive analyses focus on first observed events within the follow-up window to characterise inequities in initial access to services.

## Methods

### Public and patient involvement and engagement (PPIE)

We consulted with PPIE groups of parents and young people to help us to shape the research, interpret the results and identify limitations of this study. This led to the inclusion of the ‘refused’ racial-ethnic category in our analysis and helped contextualise findings on non-linkage in relation to opt-outs among some minoritised ethnic groups. These groups explored the social determinants of service use and informed the inclusion of additional groups, including persistently absent pupils.

### Datasets and linkage

This study used the ‘Education and Child Health Insights from Linked Data’ (ECHILD) database, which contains individually linked and de-identified education, social care and health records for pupils in England.^16,17^ The data sources used in this study were the National Pupil Database (NPD), Hospital Episode Statistics (HES), the Mental Health Services Data Set (MHSDS) and Office for National Statistics (ONS) mortality records. NPD contains information about state-funded schools in England, including school enrolments, pupil characteristics, recorded SEND provision, attainment, absences and exclusions. NPD also contains the ‘Children Looked After’ return, relating to children in care. HES contains administrative, demographic and clinical information on inpatient admissions (admitted patient care records), outpatient appointments and ED attendance activity (the Emergency Care Data Set, ECDS). MHSDS contains referrals to and contacts with wholly or partially NHS-funded mental health services. ONS mortality records provide information on the cause and date of deaths registered in England and Wales, including those that occur outside of hospital.^18,19^

These datasets were linked by NHS England using a two-stage deterministic linkage algorithm using natural identifiers (forename, surname, sex, date of birth and postcode), with reported linkage rates of 98% between NPD and HES for a reference cohort of children born in 2004/05.^16^ Linkage rates for the present study cohort (overall 95.5%) are reported in the Results and in online supplemental table S6. To be eligible to be linked to health datasets, pupils were required to have had any NHS contact (eg, registration with a general practice) and to not have opted out of sharing their data for research through the national data opt-out programme.^20^ Online supplemental table S1 gives further details of the ECHILD datasets used in this study.

### Study design

Secondary school cohort study (see online supplemental figure S1).

### Population

Our population was defined as pupils enrolled in year 7 of state-funded secondary school in England between 2012/13 and 2021/22, corresponding to age 11 years at entry (online supplemental figure S1). Enrolment was defined as a record in the January School Census, alternative provision or pupil referral unit census. For pupils not following the national curriculum (including some pupils receiving SEND provision), school year was assigned based on their age at entry (ie, age 11 years for year 7). We excluded pupils (i) who did not link to the health datasets, including those with national data opt-outs applied to their health records;^21^ (2) aged <10 or >12 years old at entry into year 7; (3) who had missing data on residential address or had a Middle Layer Super Output Area indicating Welsh/Scottish residence. The implications of these exclusions, and the restriction to state-funded school enrolment (thereby excluding privately educated pupils), are discussed with respect to generalisability in the Strengths and Limitations section.

### Follow-up

Start of follow-up differs by outcome (see online supplemental figure S1). For hospital admissions, follow-up begins from entry into year 7. Follow-up for mental health services and ED attendances reflects entry into year 7 after 1 September 2016 for MHSDS and 1 September 2020 for ECDS, as data are not available at earlier timepoints. For all outcomes, follow-up ends at the first chronological event of outcome event, death, age 21 years or end of study (31^st^ August 2022). Only the first observed event of each outcome type is captured within the follow-up window, rather than all events. This approach characterises inequities in initial access to services rather than intensity of service use and avoids rates being skewed by repeated contacts from the same individuals.

### Social strata

Social strata are (where possible) aligned with NHS England’s Core20PLUS5 target populations, and the theoretical pathways between social strata and healthcare contacts are shown in online supplemental figure S2.^14^ We use the term social strata to mean the different positions occupied by individuals in society based on their socially constructed identities and socioeconomic circumstances.^22^ We included: parent-reported gender (female or male, only two options available); region of residence (nine groups mapped to middle layer super output area of the child’s residential address); index of multiple deprivation (IMD) quintiles based on residential address; free school meal (FSM) eligibility (yes or no); and racial-ethnic group (derived from the parent-reported 20-category minor variable). We use the term “racial-ethnic” to signify that this variable contains both race-based (eg, White, Black) and ethnicity-based (eg, British, Caribbean) identifiers. Social strata were defined at the January Census of year 7 (or the nearest prior non-missing Spring Census). Gender and racial-ethnic categories are derived from parent-reported data recorded in the School Census, rather than from clinical or administrative assignment.

### High-risk populations

We included a secondary group of circumstances that are associated with, or indicative of, poorer mental health in adolescence to provide currently lacking information on groups of adolescents in high need of mental health support: care experienced (yes or no), defined as a child who has been in the care of their local authority for more than 24 hours in the 5 years prior to study entry (between school years 1–6, ages 5/6–10/11 years); chronic conditions, identified using the Hardelid code list applied to hospital admissions in the 5 years prior to study entry (school years 1–6; see online supplemental appendix 1 for codes and definitions)^23^; recorded SEND provision (SEND support, education, health and care plans) in NPD at entry into the cohort; and persistent absence, defined as ≥10% missed sessions in year 6, or year 4 and year 5 for the 2021/22 and 2020/21 cohorts, respectively (as absence data is not available for 2019/20-2020/21 due to the COVID-19 pandemic).

### Covariates

We stratified results by age at outcome event (11–<16 years, 16–<18 years, 18–<21 years) due to differences in mental health presentations, referral pathways, and thresholds for hospital admissions by age.^24,25^ These age bands align with key transitions in education and service eligibility, including the typical age range for CAMHS/CYPMHS provision (11–15 years), the transition from child to adult mental health services (around age 18), and the transition out of compulsory schooling. Age at outcome event was calculated as date of the outcome event minus the 15th day of the child’s month and year of birth (as full date of birth is not available in ECHILD).

### Outcomes

We examined three mental health-related outcomes: referrals to mental health services, emergency hospital admissions and ED attendances.

#### Referrals to NHS-funded mental health services

We captured rates of referrals to NHS-funded mental health services, including children and adolescent mental health services (CAMHS, also known as CYPMHS), using MHSDS. Given the focus on mental health, referrals recorded as relating solely to neurodevelopmental concerns or neurodiversity-related difficulties were excluded (online supplemental table S2). We report proportions of all and “crisis” (only) referral rates by social strata and high-risk groups (online supplemental table S2).

#### Mental health-related ED attendances

We defined mental health-related ED attendances as any ECDS record during the follow-up period with a primary diagnosis/chief complaint code appearing in either of two NHS England Systematised Nomenclature of Medicine Clinical Terms (SNOMED CT) code lists for psychology/toxicology/drug and alcohol or psychosocial/behaviour changes such as self-harm (online supplemental table S3).^26^ This approach may under-ascertain cases where mental health concerns are present but not recorded in the primary field.

#### Mental health-related emergency hospital admissions

We defined mental health-related emergency hospital admissions as a hospital admission during the follow-up period with an emergency admission method in the first episode of care; and International Classification of Diseases 10th Revision (ICD-10) code(s) recorded in any diagnosis field indicating mental disorder or adversity-related injury (online supplemental table S4).^27^ In sensitivity analyses, we also included potentially psychosomatic symptoms in our definition of emergency hospital admissions (online supplemental table S5).^25^

### Statistical analyses

We described the characteristics of our study population, including missing data, in numbers and percentages. Observed incidence rates of referrals to mental health services, hospital admissions and ED attendances for each social strata and high-risk population group were calculated by dividing the number of events by person-time at risk per 1000 person-years. We fitted separate Poisson regression models for each outcome, stratified by social strata and age band, including the logarithm of person-time as the offset. Follow-up time was split by calendar year and by age band, and Poisson regression with a person-time offset was fitted to the resulting time-split data. Stratification by age band was necessary to account for known differences in mental health needs and presentations by age.^15,25,28^ We adjusted for follow-up year to account for likely period effects, including the COVID-19 pandemic.^15,29^ School identifiers were not available and standard errors were estimated at pupil-level using the Huber/White sandwich estimator.

We estimated year-adjusted incidence rates (per 1000 person-years) for each social stratum and age group. We further estimated incidence rate ratios (IRRs) comparing outcomes within social strata and age groups, using the observation-weighted grand mean predicted rate as the reference group for ratios to avoid delineating non-reference groups as “other”.^30^ In additional analyses, we further stratified models by gender to explore intersectional experiences of social identities.^22^ Statistical analyses were conducted using Stata v18.0 in the ONS Secure Research Service. The code and algorithms used in this study are available from: https://github.com/UCL-CHIG/Mental_health_contacts. We made some minor deviations from our study protocol based on data availability at the time of analysis, including reducing the study end date by a year and not reporting mental health service waiting times.^31^

## Results

### Cohort derivation and characteristics

There were 5 920 062 pupils enrolled in year 7 of state-funded secondary school in England between 2012/13 and 2021/22 (online supplemental figure S3). We excluded 28 599 (0.5%) pupils with a missing or non-English residential address or who were aged <10 or >12 years at entry into year 7. We further excluded 266 716 (4.5%) pupils who did not have a linked health record. Non-linkage rates were particularly high among pupils from Black Caribbean (11 451/72 558; 15.8%), Black (other) (4136/42 063; 9.8%) and ‘refused’ (3867/43 337; 8.9%) racial-ethnic groups, as well as those with a residential address in London (61 990/879 271; 7.1%), thereby indicating non-linkage as a potential driver of selection bias; the implications of this differential non-linkage for between-group comparisons are discussed in the Strengths and Limitations section (online supplemental table S6). Finally, we excluded 13 pupils who had a death recorded before estimated entry into year 7. The final cohort included 5 629 720 pupils, of whom 51.3% (2 886 323) were male, 69.6% (3 917 140) were White British and 30.4% (1 712 435) had a residential address in London or the South East (table 1).

**Table 1:** Final cohort characteristics and unadjusted rates of mental health-related contacts across service types

	Final cohort	Rate per 1000 person-years (95% CI)			
	N (%)	Mental health service referrals	Emergency department attendances	Hospital admissions	Hospital admissions (including psychosomatic presentations)
Total	5 629 720 (100.0)	45.2 (45.1, 45.2)	13.9 (13.8, 14.0)	4.1 (4.1, 4.1)	10.0 (9.9, 10.0)
Gender					
Male	2 886 323 (51.3)	35.7 (35.6, 35.8)	8.2 (8.2, 8.3)	1.8 (1.8, 1.8)	6.6 (6.5, 6.6)
Female	2 743 397 (48.7)	55.5 (55.4, 55.7)	19.9 (19.8, 20.1)	6.5 (6.5, 6.5)	13.6 (13.6, 13.7)
Region of residence*					
East Midlands	493 995 (8.8)	43.6 (43.3, 43.9)	15.0 (14.7, 15.3)	3.7 (3.6, 3.8)	8.8 (8.7, 8.9)
East of England	633 420 (11.3)	46.6 (46.3, 46.9)	13.1 (12.9, 13.4)	3.4 (3.4, 3.5)	9.1 (9.0, 9.2)
London	817 255 (14.5)	37.7 (37.5, 37.9)	11.5 (11.3, 11.7)	2.7 (2.6, 2.7)	7.4 (7.3, 7.5)
North East	271 510 (4.8)	58.2 (57.7, 58.7)	15.4 (15.0, 15.7)	5.0 (4.9, 5.1)	12.3 (12.1, 12.5)
North West	769 030 (13.7)	48.2 (47.9, 48.4)	15.7 (15.4, 15.9)	5.1 (5.0, 5.1)	12.2 (12.1, 12.3)
South East	895 175 (15.9)	47.4 (47.1, 47.6)	13.9 (13.7, 14.1)	4.4 (4.3, 4.4)	9.8 (9.7, 9.9)
South West	533 180 (9.5)	43.3 (43.0, 43.5)	13.4 (13.2, 13.6)	5.4 (5.4, 5.5)	11.7 (11.6, 11.8)
West Midlands	634 915 (11.3)	46.3 (46.0, 46.6)	14.2 (13.9, 14.4)	4.2 (4.1, 4.2)	11.1 (11.0, 11.2)
Yorkshire and the Humber	581 235 (10.3)	42.9 (42.6, 43.2)	14.2 (14.0, 14.5)	3.5 (3.5, 3.6)	9.1 (9.0, 9.2)
IMD groups					
1 Most deprived 20%	1 409 715 (25.0)	49.8 (49.7, 50.0)	15.5 (15.3, 15.6)	4.5 (4.4, 4.5)	11.0 (11.0, 11.1)
2	1 141 097 (20.3)	48.4 (48.1, 48.6)	15.3 (15.1, 15.4)	4.4 (4.3, 4.4)	10.5 (10.4, 10.5)
3	1 031 097 (18.3)	45.5 (45.3, 45.7)	13.8 (13.6, 14.0)	4.1 (4.1, 4.2)	10.1 (10.0, 10.2)
4	987 288 (17.5)	42.2 (42.0, 42.4)	12.7 (12.5, 12.8)	3.8 (3.7, 3.8)	9.3 (9.2, 9.4)
5 Least deprived 20%	1 060 525 (18.8)	38.3 (38.1, 38.5)	11.5 (11.4, 11.7)	3.5 (3.4, 3.5)	8.6 (8.5, 8.7)
FSM eligible					
No	4 612 964 (81.9)	39.5 (39.4, 39.6)	12.4 (12.3, 12.5)	3.6 (3.6, 3.6)	9.2 (9.1, 9.2)
Yes	1 016 756 (18.1)	75.3 (75.0, 75.6)	20.8 (20.6, 21.0)	6.6 (6.5, 6.6)	14.0 (13.9, 14.1)
Racial-ethnic group					
Bangladeshi	93 044 (1.7)	23.8 (23.3, 24.3)	7.9 (7.5, 8.3)	2.2 (2.0, 2.3)	7.2 (6.9, 7.4)
Indian	159 566 (2.8)	16.7 (16.4, 17.1)	5.9 (5.7, 6.2)	1.4 (1.4, 1.5)	6.1 (5.9, 6.2)
Asian (other)	97 113 (1.7)	19.8 (19.4, 20.3)	7.5 (7.1, 7.9)	1.9 (1.8, 2.0)	6.2 (5.9, 6.4)
Pakistani	237 384 (4.2)	20.8 (20.5, 21.1)	7.1 (6.8, 7.3)	1.8 (1.7, 1.9)	8.8 (8.7, 9.0)
Black African	203 440 (3.6)	19.5 (19.2, 19.8)	8.5 (8.2, 8.8)	1.8 (1.7, 1.8)	6.2 (6.1, 6.4)
Black Caribbean	61 098 (1.1)	43.7 (42.9, 44.6)	13.4 (12.7, 14.1)	3.0 (2.8, 3.2)	7.9 (7.6, 8.2)
Black (other)	37 928 (0.7)	30.8 (30.0, 31.7)	11.4 (10.6, 12.2)	2.7 (2.5, 3.0)	7.4 (7.1, 7.8)
Chinese	21 878 (0.4)	18.4 (17.5, 19.3)	6.0 (5.3, 6.8)	1.6 (1.4, 1.9)	4.0 (3.7, 4.4)
Mixed (other)	110 034 (2.0)	46.3 (45.7, 47.0)	14.4 (13.8, 14.9)	4.4 (4.2, 4.6)	9.4 (9.1, 9.7)
Mixed Asian & White	74 262 (1.3)	44.7 (43.9, 45.5)	14.1 (13.5, 14.8)	4.5 (4.3, 4.8)	9.6 (9.3, 10.0)
Mixed Black African & White	41 962 (0.8)	49.1 (48.0, 50.2)	16.8 (15.9, 17.7)	4.6 (4.3, 4.9)	9.7 (9.2, 10.1)
Mixed Black Caribbean & White	84 974 (1.5)	64.5 (63.7, 65.5)	19.6 (18.9, 20.3)	5.6 (5.3, 5.8)	11.4 (11.1, 11.7)
Other	98 276 (1.8)	26.6 (26.1, 27.1)	8.9 (8.5, 9.3)	2.3 (2.1, 2.4)	7.1 (6.9, 7.4)
Refused	39 470 (0.7)	44.0 (42.9, 45.1)	13.2 (12.4, 14.1)	4.3 (4.0, 4.6)	9.2 (8.8, 9.7)
White British	3 917 142 (69.6)	52.0 (51.9, 52.1)	15.6 (15.5, 15.7)	4.6 (4.6, 4.7)	10.8 (10.8, 10.9)
White Irish	16 621 (0.3)	47.5 (45.9, 49.2)	15.1 (13.7, 16.5)	4.3 (3.9, 4.7)	9.8 (9.2, 10.5)
White Irish Traveller	3301 (0.1)	70.8 (66.2, 75.8)	18.8 (15.6, 22.6)	5.3 (4.3, 6.5)	12.9 (11.3, 14.8)
White (other)	311 404 (5.5)	30.4 (30.1, 30.7)	10.2 (9.9, 10.4)	2.9 (2.9, 3.0)	7.7 (7.5, 7.8)
White Romany Gypsy	17 084 (0.3)	33.8 (32.4, 35.2)	10.5 (9.4, 11.7)	3.1 (2.7, 3.5)	9.5 (8.9, 10.2)
Missing	3739 (0.1)	29.8 (27.1, 32.7)	10.6 (8.4, 13.4)	3.2 (2.5, 4.1)	7.6 (6.5, 9.0)
Chronic condition					
No	5 232 296 (92.9)	43.3 (43.2, 43.4)	13.6 (13.5, 13.6)	3.9 (3.9, 4.0)	9.4 (9.4, 9.4)
Yes	397 424 (7.1)	71.7 (71.2, 72.1)	18.3 (18.0, 18.7)	5.8 (5.7, 5.9)	17.9 (17.7, 18.1)
Recorded SEND provision					
None	4 610 711 (81.9)	38.5 (38.4, 38.6)	13.0 (12.9, 13.0)	3.8 (3.8, 3.9)	9.5 (9.4, 9.5)
SEND support	802 932 (14.3)	71.9 (71.6, 72.2)	18.9 (18.6, 19.1)	5.4 (5.3, 5.5)	12.3 (12.2, 12.4)
EHCP	216 077 (3.8)	105.9 (105.1, 106.7)	15.7 (15.3, 16.1)	4.2 (4.1, 4.4)	11.3 (11.1, 11.5)
Persistent absences (year 6)†					
No	5 097 429 (90.5)	42.5 (42.4, 42.6)	13.3 (13.2, 13.4)	3.8 (3.8, 3.9)	9.4 (9.4, 9.4)
Yes	400 479 (7.1)	87.6 (87.2, 88.1)	23.1 (22.7, 23.4)	7.3 (7.1, 7.4)	18.1 (17.9, 18.2)
Missing	131 812 (2.3)	30.2 (29.8, 30.7)	9.8 (9.4, 10.2)	3.0 (2.8, 3.1)	7.8 (7.6, 8.0)
Care experienced‡					
No	5 573 320 (99)	44.4 (44.3, 44.5)	13.6 (13.6, 13.7)	4.0 (4.0, 4.0)	9.9 (9.8, 9.9)
Yes	56 400 (1)	141.9 (140.2, 143.7)	41.2 (39.9, 42.5)	13.0 (12.6, 13.5)	19.8 (19.3, 20.4)

* Counts are rounded to the nearest 5 as per statistical disclosure rules for sub-national numbers.

† Year 4 and year 5 for the 2021/22 and 2020/21 cohorts, respectively (as absence data is not available for 2019/20-2020/21 due to the COVID-19 pandemic).

‡ Counts are rounded to the nearest 10 and percentages to the nearest whole number as per statistical disclosure rules for data from the Children Looked After dataset.

FSM, free school meal; IMD, index of multiple deprivation; SEND, special educational needs and disability.

### Referrals to mental health services

Across all ages during follow-up (mean=3.86 years, SD=1.95), 17.4% (980 648) of adolescent pupils had a recorded referral to mental health services, a rate of 45.2 per 1000 person-years (95% CI 45.1 to 45.2; table 1). Overall, 1.8% (17,486) of these referrals met our definition for crisis referral, online supplemental table S7. There was marked variation in unadjusted rates of referrals by social strata (table 1). Females (55.5, 95% CI 55.4 to 55.7) had higher observed rates of referrals than males (35.7, 95% CI 35.6 to 35.8). Within region, observed rates were highest in the North East (58.2, 95% CI 57.7 to 58.7) and lowest in London (37.7, 95% CI 37.5 to 37.9). There was a gradient in referral rates by IMD quintiles, from 38.3 (95% CI 38.1 to 38.5) among pupils living in the least deprived to 49.8 (95% CI 49.7 to 50.0) in the most deprived areas. Pupils who were FSM eligible had the highest observed rates of referrals of any social strata studied (75.3, 95% CI 75.0 to 75.6). Of the racial-ethnic groups, the lowest rates of referrals were observed among the Indian and Black African groups and the highest among the mixed Black Caribbean-White and White Irish Traveller groups. All high-risk populations had higher observed rates of referrals than average, including pupils with care experience (141.9, 95% CI 140.2 to 143.7), those with a recorded EHCP (105.9, 95% CI 105.1 to 106.7), with persistent absence in year 6 (87.6, 95% CI 87.2 to 88.1) and hospital recorded chronic conditions (71.7, 95% CI 71.2 to 72.1). Unadjusted rates of first crisis referral followed similar stratum-specific patterns to all referrals, aside from region, where the South West, West Midlands and South East had lower rates than London (online supplemental table S7).

When grouped by age, stratum-specific rates by gender, region, IMD and FSM eligibility showed consistent patterning, with higher rates among the youngest age groups (11–<16 years; figure 1; online supplemental table S8). In contrast, several racial-ethnic groups had similar rates among 11–<16 and 16–<18-year-olds; Bangladeshi, Indian, Asian (other), Pakistani, Black African, Black (other), Chinese, Other, ‘refused’, White Irish Traveller, White Romany Gypsy and those with missing data on racial-ethnic group. Relative differences in rates (IRRs; online supplemental figure S4 and table S9) highlight that (relative) differences between racial-ethnic groups tended to reduce with increasing age. Further stratification by gender for the 11–<16-year-old group (online supplemental figure S5; rates and online supplemental figure S6; IRRs) highlights the overlap in rates for females and males from White Irish Traveller and White Romany Gypsy racial-ethnic groups.

**Figure 1:**
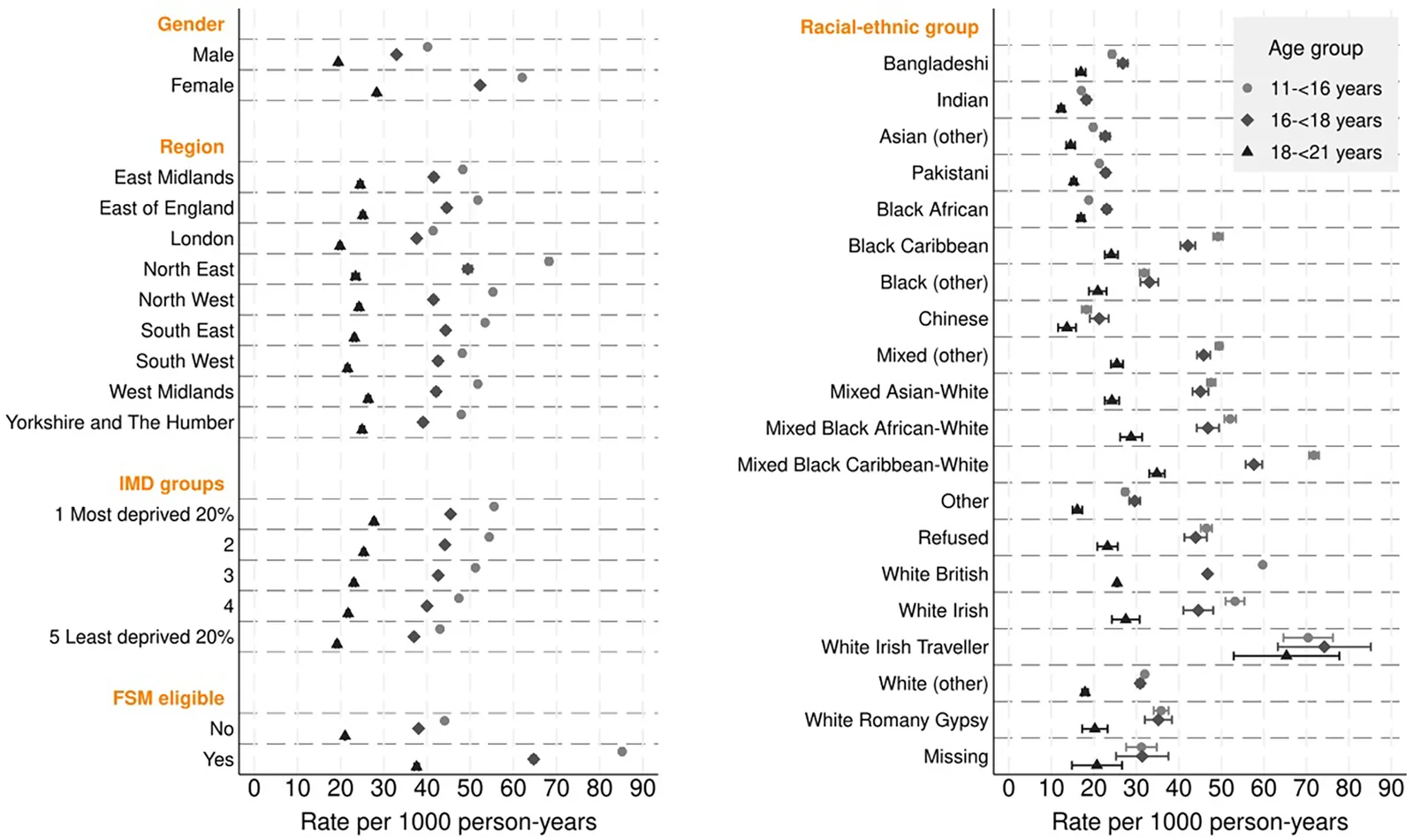
Year-adjusted rates of first referral to NHS-funded mental health services (per 1000 person-years) with 95% CIs: derived from age group-specific Poisson regression models for each group of social strata. FSM, free school meals; IMD, index of multiple deprivation.

### Mental health-related ED attendances

ED attendances were captured from September 2020 onwards (when ECDS data first became available), yielding a shorter observation window relative to referrals and admissions (mean follow-up of 1.82 years for ED attendances, 3.86 years for mental health service referrals and 5.18 years for hospital admissions). Across all ages during follow-up (mean=1.82 years, SD=0.39), 2.5% (142 118) of pupils had a recorded mental health-related ED attendance, a rate of 13.9 per 1000 person-years (95% CI 13.8 to 14.0; table 1). Variation in unadjusted rates of referrals by gender was marked (table 1), with females (19.9, 95% CI 19.8 to 20.1) having more than double the observed rates of males (8.2, 95% CI 8.2 to 8.3). Within region of residence, rates were highest in the North West (15.7, 95% CI 15.4 to 15.9) and lowest in London (11.5, 95% CI 11.3 to 11.7). A gradient in rates was present across IMD groups. Pupils who were FSM-eligible had the highest observed attendance rate of any social strata studied (20.8, 95% CI 20.6 to 21.0). Across racial-ethnic groups, rates were lowest among Indian and Chinese pupils and highest among Mixed White-Black Caribbean and White Irish Traveller pupils (online supplemental table S10 outlines full stratum-specific figures). All high-risk populations had higher rates than the cohort average, particularly care-experienced pupils (41.2, 95% CI 39.9 to 42.5).

When grouped by age, stratum-specific rates broadly increased with age, with some overlap in 95% CI for the two older age groups (figure 2; online supplemental table S10). Relative differences (IRRs) in rates were particularly high among females age 11-<16 years compared with the other age groups (online supplemental figure S7 and table S11). Further stratification by gender for the 11-<16 year old group in online supplemental figure S8 (rates) and online supplemental figure S9 (IRRs) indicated particularly low rates for Chinese females.

**Figure 2:**
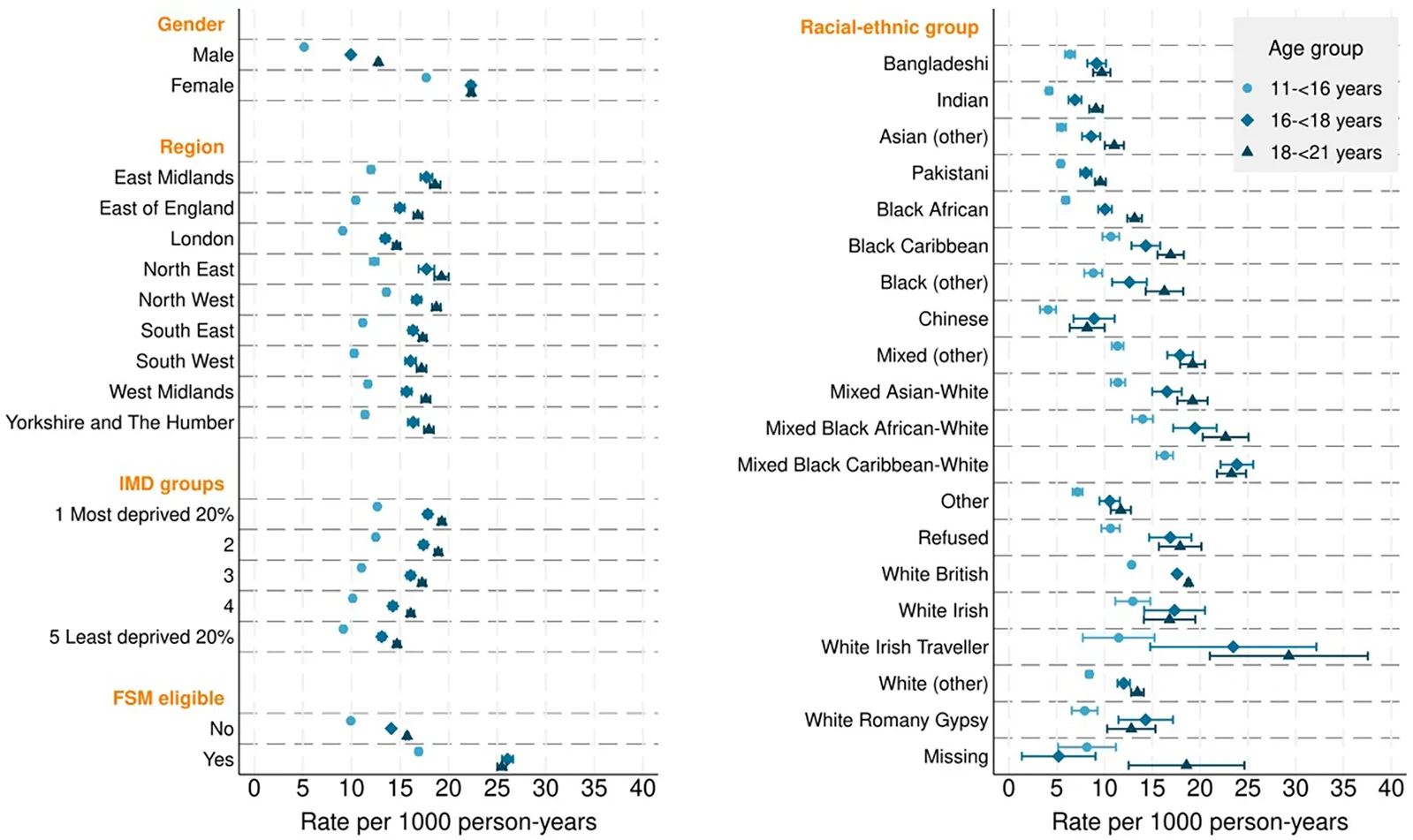
Year-adjusted rates of first mental health-related emergency department attendance (per 1000 person-years) with 95% CIs: derived from age group-specific Poisson regression models for each group of social strata. FSM, free school meals; IMD, index of multiple deprivation.

### Mental health-related emergency hospital admissions

Across all ages during follow-up (mean=5.18 years, SD=2.78), 2.1% (118,680) of pupils had a recorded mental health-related emergency hospital admission, a rate of 4.1 per 1000 person-years (95% CI 4.1 to 4.1; table 1). Females (6.5, 95% CI 6.5 to 6.5) had more than three times the observed rates of males (1.8, 95% CI 1.8 to 1.8). Within region, rates were highest in the South West (5.4, 95% CI 5.4 to 5.5) and lowest in London (2.7, 95% CI 2.6 to 2.7). A gradual social gradient was estimated across IMD groups. Pupils who were FSM eligible had the highest observed rates of admissions of any social strata studied (6.6, 95% CI 6.5 to 6.6). Across racial-ethnic groups, rates were again lowest among Indian and Chinese pupils and highest among Mixed White-Black Caribbean and White Irish Traveller pupils (full figures in online supplemental table S12). High-risk populations all had higher rates than the cohort average, with care-experienced pupils (13.0, 95% CI 12.6 to 13.5) and pupils with persistent absence (7.3, 95% CI 7.1 to 7.4) the most elevated.

When grouped by age, stratum-specific admission rates were not consistently patterned (figure 3; online supplemental table S12): rates increased with age for males but were lowest in the 18-<21-year-old group for females. Regional rates were similar across age-groups aside from the South East and South West, where the 16-<18-year-old group had the highest rates. Rates were similar across age groups for racial-ethnic groups, apart from the Asian (other), Pakistani and Black African groups, who had lower rates in the youngest age group. Relative differences (IRRs) in rates are indicated in online supplemental figure S10 and table S13, with further stratification by gender for the 11-<16 year old group in online supplemental figure S11 (rates) and online supplemental figure S12 (IRRs). These indicate that relative rates within-gender were similar across all strata aside from Bangladeshi (higher in males) and White (other) (higher in females) pupils.

**Figure 3:**
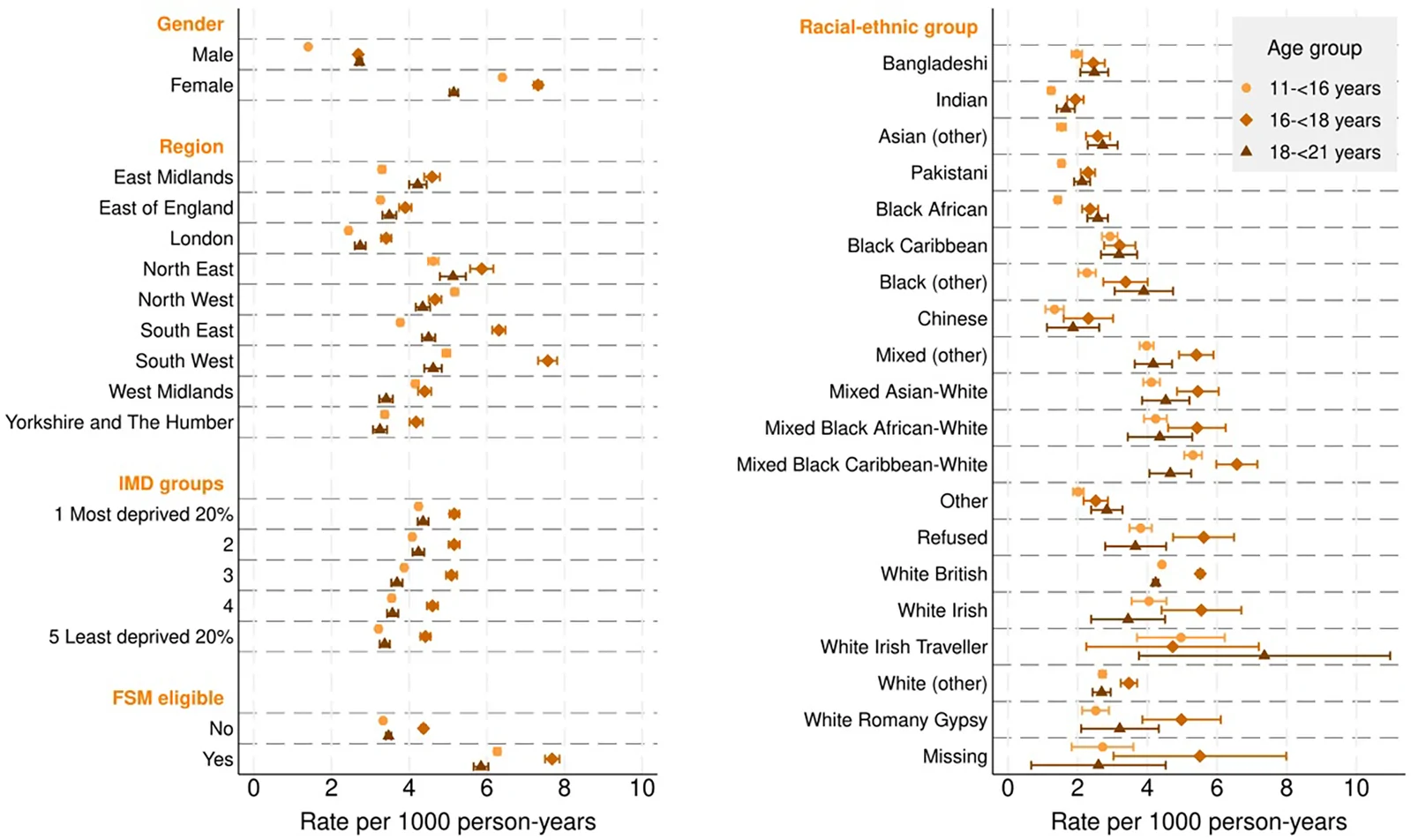
Year-adjusted rates of first mental health-related emergency hospital admissions (per 1000 person-years) with 95% CIs: derived from Poisson regression models with separate models for each social strata and age group. FSM, free school meals; IMD, index of multiple deprivation.

Adding potentially psychosomatic symptoms into the definition of mental health-related emergency hospital admissions increased the overall rate of admissions to 10.0 per 1000 person-years (95% CI 9.9 to 10.0), table 1, but generally similar patterning by strata remained (online supplemental figure S13 and table S14). Relative measures (by age group; online supplemental figure S14 and table S15) indicated that the broader case definition for admissions typically resulted in shifting stratum-specific IRRs that were closer to the mean. This shift towards the mean was particularly pronounced for Pakistani pupils aged 11-<16 years (from an IRR of 0.43 (95% CI 0.41 to 0.45) to 0.92 (95% CI 0.90 to 0.94)).

#### Combined relative results (11–<16 year old age group only)

Figure 4 displays the relative results across all three service contact points for the youngest age group. This highlights qualitatively similar relative differences within social strata. For example, IRRs of less than one for most Asian groups (Bangladeshi, Indian, Asian (other), Pakistani, Chinese) and IRRs at or above one for Mixed groups (Mixed (other), Mixed Asian-White, Mixed Black African, Mixed Black Caribbean-White), White British, White Irish and White Irish Traveller groups. The one exception is region; pupils living in the East Midlands had higher relative rates of ED attendances than average and those living in the South West had higher relative rates of hospital admissions (but lower relative rates for other services).

**Figure 4:**
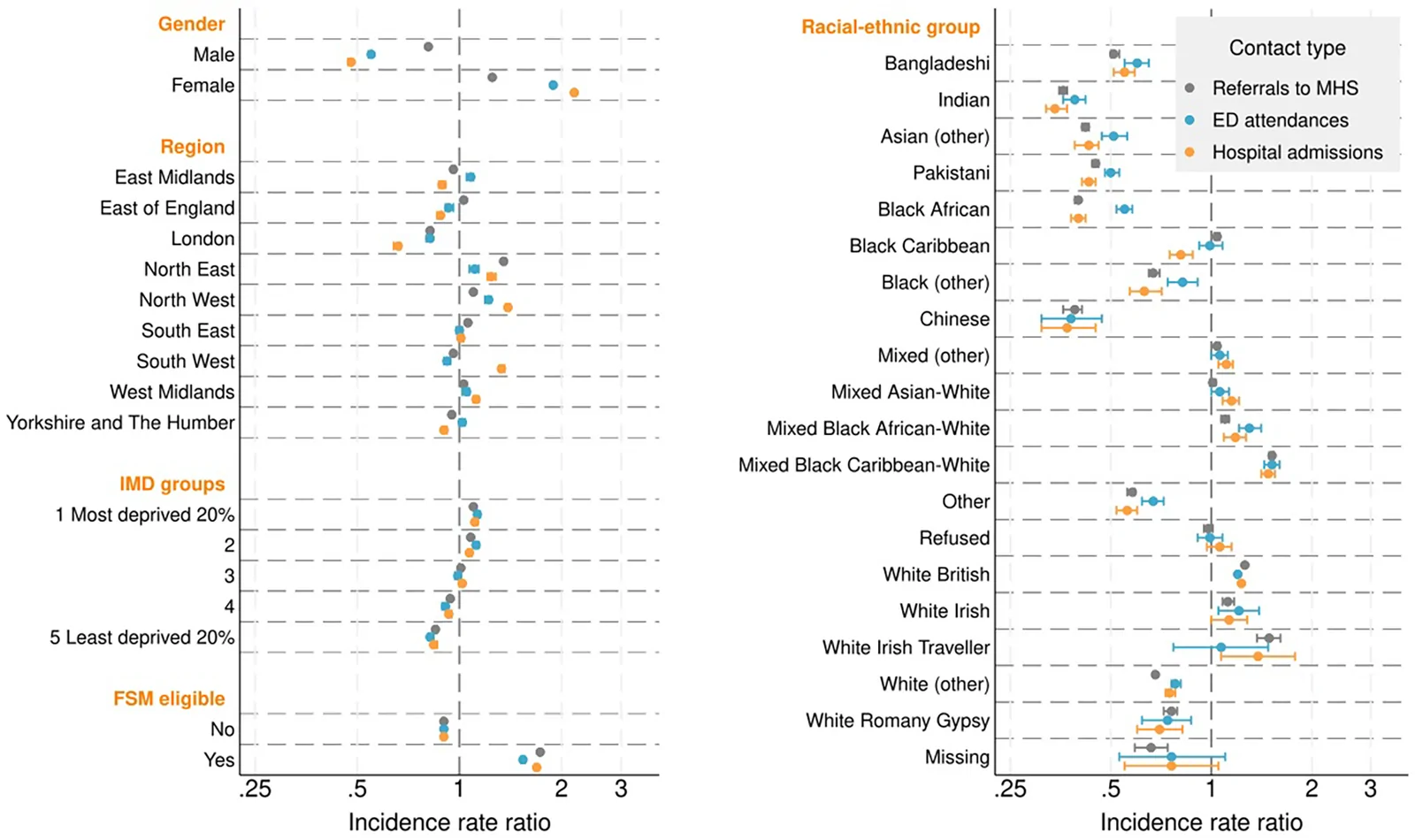
Year-adjusted incidence rate ratios (compared with the social strata- and service-specific mean rate) of first referrals to mental health services, mental health-related emergency department attendances and emergency hospital admissions: derived from service-specific Poisson regression models with separate models for each social strata (11-<16 years old group only). ED, emergency department; FSM, free school meals; IMD, index of multiple deprivation; MHS, mental health services. Note. Follow-up windows differ across the three outcomes: hospital admissions were observed from 2012/13 onwards (mean follow-up 5.18 years), mental health service referrals from 2016/17 onwards (mean follow-up 3.86 years), and ED attendances from September 2020 onwards (mean follow-up 1.82 years). The differing windows mean these cross-service comparisons reflect cohorts exposed to different levels of pandemic disruption and policy change; see Discussion (Interpretation and implications) for interpretation.

## Discussion

In this national cohort study of secondary school pupils in England, we find clear inequalities in rates of mental health-related service contacts by social strata. Notably, higher rates of referrals to mental health services, ED attendances and hospital admissions were shown for females, pupils who were FSM eligible, in the most deprived IMD groups, living in the North, White Irish Travellers and Mixed Black Caribbean-White. Consistently lower rates were estimated for males, pupils living in London and several racial-ethnic groups, including Bangladeshi, Indian, Pakistani and Black African. All high-risk groups had higher than average rates, particularly care-experienced pupils who had three times the average rate for referrals to mental health services and ED attendances. There was consistency in relative rates across services, particularly for socioeconomic circumstances (IMD and FSM eligibility) and racial-ethnic groups, likely reflecting systematic differences underpinned by social determinants. However, service contact data alone cannot disentangle differences in underlying need from differences in help-seeking, referral pathways, service availability and recording.

### Strengths and limitations

To our knowledge, this is the first whole-nation secondary school cohort to look at patterns of mental health contacts across multiple NHS services, which is a critical gap given rising rates of urgent and emergency healthcare for adolescent mental health.^15^ The large population supported granular analysis of important population groups. Investigating three health contact points provides a richer understanding of social and service-level inequalities in contact. In follow-up work, findings from this study will be triangulated with evidence on young people’s mental health service use in Bradford, which includes an indicator of mental health need to better understand the implications for inequities in service contact relative to underlying need.^31^

Due to data quality concerns, we focused on referrals to mental health services (a mandatory field in MHSDS) as an indicator of perceived need paired with healthcare seeking and reaching (but not care received). A strength of our study is the use of standardised code lists developed for ECDS submissions.^32^ However, ECDS was only available for 2 years of our study (2020/21 and 2021/22) and presentations before that time are not captured in this study. The availability of ECDS data overlaps with the COVID-19 lockdown,^33,34^ during which time ED attendance rates were lower and recorded using a limited list of SNOMED codes. Thus ED mental health-related events may be under-ascertained. Period effects, including those associated with the COVID-19 pandemic, are captured through adjustment for follow-up year in all models.

Only pupils who enrolled in year 7 of state-funded school during the study period are included in our cohort, with the implication that the findings are not generalisable to young people who never attend state-funded secondary school in England. Alongside home schooling and exclusion, the most common reason for not attending a state-funded school is attendance at a fee-paying school (6–7% of the population),^35^ meaning our results likely under-represent pupils from advantaged backgrounds. Home-schooled pupils, those educated outside the school census entirely, and those who left state-funded education before year 7, are also not represented in our cohort.

In total, 4.5% of pupils were not linked to the health datasets and were excluded from this study. Linkage was non-random and varied substantially by racial-ethnic group (higher in all minoritised ethnicities, particularly among Black Caribbean pupils). Non-links can occur for several reasons, including no contact with the NHS, missed links due to inconsistently recorded personal information and data opt-outs (accounting for 87% of non-links across ECHILD).^36^ Non-linkage rates were 15.8% among Black Caribbean pupils and 7.1% among pupils resident in London (online supplemental table S6), substantially higher than the 4.5% overall figure. The direction of bias is uncertain as we cannot differentiate non-linkage due to opt-out vs no health service-use. We also excluded pupils with missing residential information, which may include populations who are at greater risk of mental ill-health, such as those experiencing housing instability.^37^

Some important factors were incompletely measured in our study, eg, sexual and gender-minoritised identity, including those with elevated mental health concerns and care barriers.^9^ The definition of care experience used here captures a heterogeneous group (eg, with respect to timing, duration and recency of care exposure). Persistent absence was measured using year 6 absence for eight of the ten entry cohorts and year 4 or year 5 absence for the 2020/21 and 2021/22 cohorts because year 6 absence data were unavailable for these cohorts due to the pandemic. This introduces some inconsistency in the persistent absence variable across the cohort.

We modelled strata separately and adjusted only for follow-up year (ie, without mutual adjustment), such that the stratum-specific estimates presented should not be interpreted as independent of other strata. For example, several minoritised racial-ethnic groups are concentrated in London and in more deprived areas; thus, the associated stratum-specific estimates for these groups draw on overlapping populations without adjustment. Social strata were measured at year 7 entry and held fixed throughout follow-up and therefore do not reflect changes over time or after leaving school (eg, FSM eligibility). A baseline-defined design also avoids capturing changes in circumstance that may themselves be downstream of mental health difficulties, for example, family disruption, housing instability or moves following crisis, which would otherwise be embedded in a time-varying specification.

Crucially, service contact rates are not equivalent to underlying mental health need, and we are therefore not able to distinguish between differences in need, symptom recognition, help-seeking, referral pathways, recording practices, and the availability and acceptability of services across population strata. The decision to analyse first observed events within the follow-up window characterises inequities in initial access to services but does not capture repeated contacts or intensity of service use, and may therefore underestimate cumulative differences in service engagement.

### Interpretation and implications

Our main finding is that there are substantial disparities in rates of health service contacts related to mental health among pupils in England. Similar associations have been observed elsewhere, including: higher rates of primary or secondary healthcare service use for emotional problems in female compared with male secondary school pupils in the Midlands and South West England^38^; higher reported contact with any professional services among White pupils with a diagnosed mental health condition compared with young people from other racial groups in a UK-based survey^24^; and a social gradient between area-level deprivation and referrals to specialist mental health services in Scotland.^39^ We find that rates of contacts for pupils who are FSM eligible are up to twice that of pupils living in the most deprived area, indicating the importance of individual-level measures for capturing the structural conditions that lead to higher levels of mental health needs.

We present rates for more granular racial-ethnic groups than typically reported, to better reflect the unique experiences of people from culturally diverse ethnic groups.^40,41^ This is particularly stark within marginalised populations usually grouped under racialised categories “White” (eg, White Irish Travellers with higher and White Romany Gypsy with lower than average rates across all service types) and “Black” (eg, Black African with near average and Black Caribbean with lower than average rates across all service types). Additional gender-stratification illustrates that gendered social risk factors are experienced differently across racial-ethnic groups. We also show that region of residence is an important source of variation in mental health-related contacts. Differences in models of service delivery across England, as well as density of services and populations, may place some populations at further risk of inequities in provision. Future research could use methods grounded in intersectional theory to describe mental health-related contacts across multiple interlocking social strata.^22^

Our results indicate that relative (but not absolute) rates across services are broadly consistent over social strata in the 11–<16 age group (figure 4), with little evidence that specific population groups disproportionately use specific service types (eg, emergency care). This is an important finding because it may suggest systematic differences in NHS service use, likely underpinned by social determinants and requiring an upstream approach that addresses both underlying health needs and barriers to access, although we caution that—due to availability—our outcome measures relate to different time periods and may therefore be subject to different external factors such as the rollout of Mental Health Support Teams.

To our knowledge, this is the first English study to examine mental health-related contacts across referrals, emergency department attendances, and emergency hospital admissions simultaneously in children and young people by social strata. This has been enabled by the recent availability of linked national datasets, particularly MHSDS and ECDS. Our findings highlight the importance of case definitions: applying a broader definition of mental health-related admissions reduced inequalities, particularly among Pakistani pupils, consistent with evidence that symptom presentation, referral route and duration of treatment vary across ethnic groups.^40,41^ Policymakers and clinicians should consider these differences when interpreting service use and making clinical decisions. Future ECHILD-based research should examine intersectional inequalities as well as transitions across services to better understand and validate mental health phenotypes in relation to clinical pathways.

## Supplementary material

10.1136/bmjph-2025-004281 online supplemental file 1

## Data Availability

Data may be obtained from a third party and are not publicly available.

## References

[1] Solmi M, Radua J, Olivola M (2022). Age at onset of mental disorders worldwide: large-scale meta-analysis of 192 epidemiological studies. Mol Psychiatry.

[2] (2024). Closing the global gap in adolescent mental health. Nat Med.

[3] National Audit Office (2023). Progress in improving mental health services in England. https://www.nao.org.uk/wp-content/uploads/2023/02/Progress-in-improving-mental-health-services-CS.pdf.

[4] Whitehead M (1992). The concepts and principles of equity and health. Int J Health Serv.

[5] Public Health England, UCL Institute of Health Equity (2017). Psychosocial pathways and health outcomes: informing action on health inequalities. https://assets.publishing.service.gov.uk/media/5a74d3e440f0b65f613228d7/Psychosocial_pathways_and_health_equity.pdf.

[6] NHS England (2023). Mental health of children and young people in England, 2023 - Wave 4 follow up to the 2017 survey. https://digital.nhs.uk/data-and-information/publications/statistical/mental-health-of-children-and-young-people-in-england/2023-wave-4-follow-up.

[7] Shim RS, Kho CE, Murray-García J (2018). Inequities in Mental Health and Mental Health Care: A Review and Future Directions. Psychiatr Ann.

[8] Meechan H, John M, Hanna P (2021). Understandings of mental health and support for Black male adolescents living in the UK. Child Youth Serv Rev.

[9] McDermott E, Eastham R, Hughes E (2024). Early mental health intervention and supported self-care for LGBTQ+ young people in the UK: a mixed-methods study. *Health Soc Care Deliv Res*.

[10] Fargas-Malet M, McSherry D (2018). The Mental Health and Help-Seeking Behaviour of Children and Young People in Care in Northern Ireland: Making Services Accessible and Engaging. Br J Soc Work.

[11] Brimblecombe N, Stevens M, Gowen S (2024). Types and aspects of support that young carers need and value, and barriers and enablers to access: the REBIAS-YC qualitative study. *Health Soc Care Deliv Res*.

[12] Majumder P, Vostanis P, Karim K (2019). Potential barriers in the therapeutic relationship in unaccompanied refugee minors in mental health. J Ment Health.

[13] Department of Health and Social Care (2025). 10 year health plan for England: fit for the future. https://www.gov.uk/government/publications/10-year-health-plan-for-england-fit-for-the-future.

[14] NHS England (2025). Core20PLUS5 – An approach to reducing health inequalities for children and young people. www.england.nhs.uk/about/equality/equality-hub/national-healthcare-inequalities-improvement-programme/core20plus5/core20plus5-cyp/.

[15] Ward JL, Vázquez-Vázquez A, Phillips K (2025). Admission to acute medical wards for mental health concerns among children and young people in England from 2012 to 2022: a cohort study. Lancet Child Adolesc Health.

[16] Mc Grath-Lone L, Libuy N, Harron K (2022). Data Resource Profile: The Education and Child Health Insights from Linked Data (ECHILD) Database. Int J Epidemiol.

[17] University College London (2024). ONS SRS metadata catalogue, dataset, education and child health insights from linked data - England.

[18] NHS Digital (2022). Linked HES-ONS mortality data. https://digital.nhs.uk/data-and-information/data-tools-and-services/data-services/linked-hes-ons-mortality-data.

[19] Office for National Statistics (2019). User guide to mortality statistics. https://www.ons.gov.uk/peoplepopulationandcommunity/birthsdeathsandmarriages/deaths/methodologies/userguidetomortalitystatisticsjuly2017.

[20] NHS England (2023). Understanding the national data opt-out. https://digital.nhs.uk/services/national-data-opt-out/understanding-the-national-data-opt-out.

[21] NHS Digital (2018). National data opt-out, December 2018. https://digital.nhs.uk/data-and-information/publications/statistical/national-data-opt-out/december-2018/december-2018.

[22] Evans CR, Leckie G, Subramanian SV (2024). A tutorial for conducting intersectional multilevel analysis of individual heterogeneity and discriminatory accuracy (MAIHDA). SSM Popul Health.

[23] Hardelid P, Dattani N, Gilbert R (2014). Estimating the prevalence of chronic conditions in children who die in England, Scotland and Wales: a data linkage cohort study. BMJ Open.

[24] Mathews F, Ford TJ, White S (2024). Children and young people’s reported contact with professional services for mental health concerns: a secondary data analysis. Eur Child Adolesc Psychiatry.

[25] Blackburn R, Ajetunmobi O, Mc Grath-Lone L (2021). Hospital admissions for stress-related presentations among school-aged adolescents during term time versus holidays in England: weekly time series and retrospective cross-sectional analysis. BJPsych Open.

[26] NHS England (2024). ECDS guidance and documents: enhanced technical output specification (ETOS) v4.0.8. https://digital.nhs.uk/data-and-information/data-collections-and-data-sets/data-sets/emergency-care-data-set-ecds/ecds-guidance.

[27] Herbert A, Gilbert R, González-Izquierdo A (2016). 10-y Risks of Death and Emergency Re-admission in Adolescents Hospitalised with Violent, Drug- or Alcohol-Related, or Self-Inflicted Injury: A Population-Based Cohort Study. PLoS Med.

[28] Pape T, Rixson L, Pari AAA (2026). Inequity in treatment access for child mental health services in England: analysis of administrative national data for 2021–2022. BJPsych Bull.

[29] Knipe D, de Ossorno Garcia S, Salhi L (2025). Digital mental health service engagement changes during Covid-19 in children and young people across the UK: Presenting concerns, service activity, and access by gender, ethnicity, and deprivation. PLoS ONE.

[30] Collaboratory for Health Justice (2021). Best practices for using race in public health research.

[31] Lewis KM, Dale H, Lewer D (2025). Adolescent mental health related contacts: ECHILD project. https://osf.io/z7e9q.

[32] NHS England (2025). ECDS guidance and documents. https://digital.nhs.uk/data-and-information/data-collections-and-data-sets/data-sets/emergency-care-data-set-ecds/ecds-guidance.

[33] Danechi S, Harker R, Stiebahl S (2025). NHS key statistics: England. https://researchbriefings.files.parliament.uk/documents/CBP-7281/CBP-7281.pdf.

[34] Robertson C, Brainard J, Smith GE (2026). Utilisation of mental health services before, during, and after COVID-19 restrictions: interrupted time-series analysis in England. BMC Health Serv Res.

[35] Independent Schools Council ISC census and annual report 2025. https://cms.isc.co.uk/media/0u0llqvv/_isc_census_2025_29may25.pdf.

[36] The UCL-ECHILD project team https://github.com/UCL-ECHILD/docs.echild.ac.uk-website/blob/main/web_source/appendix-linkage.qmd#L114.

[37] Hock ES, Blank L, Fairbrother H (2024). Exploring the impact of housing insecurity on the health and wellbeing of children and young people in the United Kingdom: a qualitative systematic review. BMC Public Health.

[38] Sayal K, Yates N, Spears M (2014). Service use in adolescents at risk of depression and self-harm: prospective longitudinal study. Soc Psychiatry Psychiatr Epidemiol.

[39] Ball WP, Black C, Gordon S (2023). Inequalities in children’s mental health care: analysis of routinely collected data on prescribing and referrals to secondary care. BMC Psychiatry.

[40] Guan S, Coughlan B, Evans K (2024). Associations between ethnicity and mental health problems among children and adolescents in the United Kingdom: A systematic review and narrative synthesis. BMC Public Health.

[41] Ní Chobhthaigh S, Jay MA, Blackburn R (2025). Emergency hospital admissions for stress-related presentations among secondary school-aged minoritised young people in England. Br J Psychiatry.

