# Supplementary material for: Disparities in adolescent mental health-related National Health Service presentations in England: a secondary school cohort study
Source: BMJ Public Health. 2026 Oct 6;4(4):e004281. doi: 10.1136/bmjph-2025-004281 (PMC13646986; doi:10.1136/bmjph-2025-004281)
Supplement: Supplementary file 1 [file bmjph-4-4-s001.docx]

# Supplementary Appendix

**Table S1.** Datasets from ECHILD used in this study

| **Dataset** | **Common acronym** | **Data provider** | **Description** | **Key variables*** | **Years** |
| --- | --- | --- | --- | --- | --- |
| **National pupil database school census** | NPD school census | Department for Education | Termly census with information for pupils in state-maintained educational settings in England, including special schools, maintained pupil referral units, alternative provision academies and free schools | Age at start of the academic year, ethnic group, gender, English as an additional language, special educational needs, MSOA | 2012/13-2022/23 |
| **NPD alternative provision census** | NPD AP census | Department for Education | Annual census with information for pupils in maintained pupil referral units, alternative provision academies and alternative provision free schools | Age at start of the academic year, ethnic group, gender, English as an additional language, special educational needs, MSOA | 2012/13-2022/23 |
| **NPD absences** | NPD absences | Department for Education | Data on the number of possible sessions and absences. Compiled from the NPD school censuses | Sessions possible, authorised absences, unauthorised absences | 2011/12-2021/22 |
| **NPD Children Looked After Return** | NPD CLA | Department for Education | National individual-level dataset, which contains information on all looked-after children and recent care leavers in England | Care episode start date, unaccompanied asylum seeker flag | 2012/13-2022/23 |
| **National Statistics**  **Postcode Lookup**** | NSPL | ONS | Opensource geographical information mapping Census Output Areas to a range of higher statistical geographies | Region, MSOA | Cross-sectional data from 2012 to 2023 |
| **Hospital episode statistics admitted patient care** | HES APC | NHS England | Episode level data on inpatient and day case discharges from English NHS hospitals and English NHS commissioned activity in the independent sector | Diagnoses, episode start data, episode end date | 2016/17-2022/23 |
| **Emergency care data set** | ECDS | NHS England | Attendances at emergency departments and urgent care in English NHS hospitals and English NHS commissioned activity in the independent sector | Attendance, admission date, discharge dates, locations, presenting complaint, diagnoses | 2020/21- 2022/23 |
| **Mental health services dataset** | MHSDS | NHS England | Holds patient-level data from the health records of children, young people and adults who are in contact in-patient, out-patient, and community mental health services | Referral rejection reason,  Referral request received date,  Service or team type referred to | 2016/17-2022/23 |
| **Mortality data** |  | ONS | Information on the cause and date of deaths registered in England and Wales (including those that occur outside of hospital). | Date of death | 2011/12-2022/23 |

*Variables as described in the source dataset; **Not an ECHILD dataset; ECHILD = Education and Child Health Insights from Linked Data; MSOA = middle super output area; NHS = National Health Service; ONS = Office for National Statistics; URN = unique reference number

 

**Figure S1.** Expected age of pupils at entry into each follow-up year: entry into study and dates of outcome datasets highlighted. ECDS = emergency care data set; HES APC = hospital episode statistics admitted patient care; MHSDS = mental health services dataset

**Figure S2.** Conceptual diagram of the social determinants of mental health needs and service access. Adapted from: Public Health England & the UCL Institute of Health Equity, 2017; Levesque, Harris & Russell, 2013

# Table S2. Mental health services dataset (MHSDS) variables and values used to define NHS-funded mental health service outcomes

| Outcome | Data dictionary name  [variable name] / MHSDS table | Description | Rules applied [values] |
| --- | --- | --- | --- |
| Referral - any | Referral request received date  [ReferralRequestReceivedDate] / MHS101 | This is the date the referral request was received by the Health Care Provider | Include referral requests received date is within specified follow-up time |
|  | Service or team type referred to  [ServTeamTypeRefToMH] / MHS102 | The type of service or team within a Mental Health Service that a patient was referred to | Include any |
| Referral - neurodevelopmental concerns/ neurodiversity-related difficulties* *** | Referral request received date  [ReferralRequestReceivedDate] / MHS101 | This is the date the referral request was received by the Health Care Provider | Include referral requests received date is within specified follow-up time |
|  | Primary reason for referral  [PrimReasonReferralMH] / MHS101 | This is the primary presenting condition or symptom for which the patient was referred to a Mental Health Service | Must only include any of:   - Neurodevelopmental conditions, excluding autism [24] - Suspected autism [25] - Diagnosed autism [26] - Behaviours that challenge due to a learning disability [30] |
|  | Service or team type referred to  [ServTeamTypeRefToMH] / MHS102 | The type of service or team within a Mental Health | Must only include any of:   - Autism services [C01] - Neurodevelopmental team [C04] - Enhanced/Intensive Support Service [E04] |
| Referral - crisis** *** | Primary reason for referral  [PrimReasonReferralMH] / MHS101 | This is the primary presenting condition or symptom for which the patient was referred to a Mental Health Service | Include: In crisis [18] |
|  | Source of referral [SourceOfReferralMH] / MHS101 | The source of referral to a Mental Health Service | Include: Acute Secondary Care: Emergency Care Department [H1] |
|  | Referral request received date  [ReferralRequestReceivedDate] / MHS101 | This is the date the referral request was received by the Health Care Provider | Include referral requests received date is within specified follow-up time |
|  | Service or team type referred to  [ServTeamTypeRefToMH] / MHS102 | The type of service or team within a Mental Health Service that a patient was referred to | Include any of:   - Crisis Resolution Team/Home Treatment Service [A02] - Psychiatric Liaison Service [A11] - 24/7 Crisis Response Line [A19] - Health Based Place Of Safety Service [A20] - Crisis Café/ SafeHaven/ Sanctuary Service [A21] - Walk-in Crisis Assessment Unit Service [A22] - Crisis House Service [A25] - Paediatric Liaison Service [C05] |

*included if matches criteria for primary reason for referral or, if reason for referral is missing, criteria for service or team type referred to; **must meet all specified criteria to be included within definition; ***linked to MHS101 (referrals table) using “uniqservreqid_deid” and “person_id_deid”; NHS = National Health Service

**Table S3.** SNOMED CT codes used to define mental health-related emergency department attendances in the Emergency Care Data Set (ECDS), by NHS England definition group

| Definition group |  | SNOMED CT Code | Description |
| --- | --- | --- | --- |
| ECDS group 1: Psych / tox / D+A* | Mental health | 33449004 | Personality disorder |
|  |  | 72366004 | Eating disorder |
|  |  | 191736004 | Obsessive-compulsive disorder |
|  |  | 371631005 | Panic disorder |
|  |  | 197480006 | Anxiety disorder |
|  |  | 35489007 | Depression |
|  |  | 13746004 | Bipolar disorder |
|  |  | 58214004 | Schizophrenia |
|  |  | 69322001 | Psychotic disorder |
|  |  | 44376007 | Dissociative disorder |
|  |  | 397923000 | Somatisation disorder |
|  |  | 30077003 | Somatoform pain disorder |
|  |  | 17226007 | Adjustment disorder |
|  |  | 50705009 | Factitious disorder |
|  | Toxicology | 295124009 | Paracetamol overdose |
|  |  | 295217003 | Non-steroidal anti-inflammatory overdose |
|  |  | 295830007 | Overdose of antidepressant drug |
|  |  | 296015009 | Sedative overdose |
|  |  | 242253008 | Overdose of opiate |
|  |  | 296335002 | Overdose of beta-adrenergic blocking drug |
|  |  | 296355001 | Overdose of calcium-channel blockers |
|  |  | 297062008 | Digoxin overdose |
|  |  | 24354007 | Poisoning by colchicine |
|  |  | 111769007 | Poisoning by antihypertensive agent |
|  |  | 419347003 | Poisoning by antibacterial drug |
|  |  | 57005003 | Poisoning by caffeine |
|  |  | 1382631000000101 | Poisoning caused by household product |
|  |  | 275390005 | Toxic effect of plant |
|  |  | 44400004 | Toxic effect of venom |
|  |  | 43302000 | Anticoagulant overdosage |
|  |  | 1148733008 | Iron and/or iron compound overdose |
|  |  | 17383000 | Toxic effect of carbon monoxide |
|  |  | 66207005 | Toxic effect of cyanide |
|  |  | 38959009 | Methaemoglobinaemia |
|  |  | 67426006 | Toxic effect of alcohol |
|  |  | 371089000 | Serotonin syndrome |
|  |  | 1148734002 | Acetylcholine receptor antagonist poisoning |
|  |  | 61356009 | Poisoning by parasympathomimetic drug |
|  |  | 371043007 | Toxic inhalation injury |
|  |  | 426936004 | Smoke inhalation injury |
|  |  | 75478009 | Poisoning |
|  | Drug / alcohol | 25702006 | Alcohol intoxication |
|  |  | 66590003 | Alcohol dependence |
|  |  | 191480000 | Alcohol withdrawal syndrome |
|  |  | 308742005 | Alcohol withdrawal-induced convulsion |
|  |  | 1098111000000101 | Drug-induced seizure |
|  |  | 307052004 | Illicit drug use |
|  |  | 268640002 | Hypnotic or anxiolytic dependence |
|  |  | 75544000 | Opioid dependence |
| Psychosocial/ behaviour change reference set** |  | 248062006 | Self-harm |
|  |  | 366979004 | Depressive feelings |
|  |  | 6471006 | Feeling suicidal |
|  |  | 48694002 | Feeling anxious |
|  |  | 248020004 | Behaviour: unusual |
|  |  | 248004009 | Behaviour: violent |
|  |  | 24199005 | Behaviour: agitation |
|  |  | 7011001 | Hallucinations |
|  |  | 2073000 | Delusions |

*Code must be in the diagnosis field with the lowest sequence number, indicating primary diagnosis; **code in the chief complaint field; NHS = National Health Service; SNOMED CT = Systematized Nomenclature of Medicine Clinical Terms.

Note: The first list is the ECDS psychology/toxicology/drug and alcohol group, including common mental health disorders, harmful effects from drugs, medicaments and other substances. Attendances were included in this definition if the code appeared in the primary diagnosis position, identified as the lowest sequence diagnosis number. The second list is the psychosocial/behaviour change reference set, which encompasses attendances with a chief complaint of self-injurious behaviour, depressed mood, suicidal thoughts, anxiety, bizarre behaviour, physical aggression, feeling agitated, hallucinations or delusions.^26^ Misclassification may arise from prioritising the primary diagnosis or chief complaint field over a broader definition that incorporates codes in secondary positions, particularly for individuals with mixed presentations.

**Table S4**. ICD-10 codes used to identify emergency hospital admissions* for mental health disorders and adversity-related injuries in hospital episode statistics admitted patient care

| Category | Sub-category | | ICD-10 codes | ICD-10 Description | |
| --- | --- | --- | --- | --- | --- |
| Mental health disorders** |  | |  |  | |
| Internalising | Mood Disorders | | F320 | Mild depressive episode | |
|  |  |  | F321 | Moderate depressive episode | |
|  |  |  | F322 | Severe depression without psychotic symptoms | |
|  |  |  | F328 | Other depressive episodes | |
|  |  |  | F329 | Depressive episode, unspecified | |
|  |  |  | F330 | Recurrent depressive disorder, current episode mild | |
|  |  |  | F331 | Recurrent depressive disorder, current episode moderate | |
|  |  |  | F332 | Recurrent depressive disorder, current episode severe without psychotic symptoms | |
|  |  |  | F333 | Recurrent depressive disorder, current episode severe with psychotic symptoms | |
|  |  |  | F338 | Other recurrent depressive disorders | |
|  |  |  | F339 | Recurrent depressive disorder, unspecified | |
|  |  |  | F341 | Dysthymia | |
|  | Anxiety or fear-related, Obsessive-compulsive, & Dissociative disorders | | F40 | Phobic anxiety disorders | |
|  |  |  | F41 | Other anxiety disorders | |
|  |  |  | F94.0 | Elective mutism | |
|  |  |  | F42 | Obsessive-compulsive disorder | |
|  |  |  | F44 | Dissociative [conversation] disorders | |
|  |  |  | F45 | Somatoform disorders | |
|  |  |  | F48 | Other neurotic disorders | |
|  |  |  | F54 | Psychological and behavioural factors associated with disorders or diseases classified elsewhere | |
|  | Disorders specifically associated with stress | | F43 | Reaction to severe stress, and adjustment disorders | |
|  |  |  | F62 | Enduring personality changes, not attributable to brain damage and disease | |
|  |  |  | F94.1 | Reactive attachment disorder of childhood | |
|  |  |  | F94.2 | Disinhibited attachment disorder of childhood | |
|  | Eating disorders | | F50 | Eating disorders | |
|  | Emotional disorders with onset usually occurring in childhood and adolescence | | F93 | Emotional disorders with onset specific to childhood | |
|  |  |  | F98 | Other behavioural and emotional disorders with onset usually occurring in childhood and adolescence | |
| Externalising | Impulse control, Disruptive behaviour or dissocial disorders | | F63 | Habit and impulse disorders | |
|  |  |  | F90 | Disturbance of activity and attention | |
|  |  |  | F91 | Conduct disorders | |
|  |  |  | F92 | Mixed disorders of conduct and emotions | |
| Thought Problems & Personality Disorders | Mood Disorders with mania or psychotic symptoms | | F30 | Manic episode | |
|  |  |  | F31 | Bipolar affective disorder | |
|  |  |  | F323 | Severe depression with psychotic symptoms | |
|  | Schizophrenia or other primary psychotic disorders | | F20 | Schizophrenia | |
|  |  |  | F21 | Schizotypal disorder | |
|  |  |  | F22 | Persistent delusional disorders | |
|  |  |  | F23 | Acute and transient psychotic disorders | |
|  |  |  | F24 | Induced delusional disorder | |
|  |  |  | F25 | Schizoaffective disorders | |
|  |  |  | F28 | Other nonorganic psychotic disorders | |
|  |  |  | F29 | Unspecified nonorganic psychosis | |
|  | Disorders of adult personality and behaviour | | F60 | Specific Personality Disorders | |
|  |  |  | F68 | Other disorders of adult personality and behaviour | |
|  |  |  | F69 | Unspecified disorder of adult personality and behaviour | |
| Adversity-related injuries*** | |  | | |  |
| Drug and/or alcohol misuse | | F11-16, F18-19 | | | Mental and behavioural disorders due to psychoactive substance use |
|  |  | R78.1- R78.5 | | | Findings of drugs and other substances, not normally found in blood |
|  |  | T36-T50 (excl T50.6) | | | Poisoning by drugs, medicaments and biological substances |
|  |  | Y10-Y14 | | | Poisoning, undetermined intent |
|  |  | Z50.3 | | | Drug rehabilitation |
|  |  | Z71.5 | | | Drug abuse counselling and surveillance |
|  |  | Z72.2 | | | Problems related to lifestyle, drug use |
|  |  | F18 | | | Mental and behavioural disorders due to use of volatile solvents |
|  |  | X40-X44 X46-X49 | | | Accidental poisoning by and exposure to noxious substances |
|  |  | X69 | | | Intentional self-poisoning by and exposure to other and unspecified chemicals and noxious substances |
|  |  | Y16-Y19 | | | Poisoning, undetermined intent |
|  |  | G40.5 | | | Special epileptic syndromes |
|  |  | Z04.0 | | | Examination for Blood-alcohol and blood-drug test |
|  |  | E24.4 | | | Alcohol-induced pseudo-Cushing syndrome |
|  |  | F10 | | | Mental and behavioural disorders due to use of alcohol |
|  |  | G31.2 | | | Degeneration of nervous system due to alcohol |
|  |  | G62.1 | | | Alcoholic polyneuropathy |
|  |  | G72.1 | | | Alcoholic polyneuropathy |
|  |  | I42.6 | | | Alcoholic cardiomyopathy |
|  |  | K29.2 | | | Alcoholic gastritis |
|  |  | K70 | | | Alcoholic liver disease |
|  |  | K85.2 | | | Alcohol-induced acute pancreatitis |
|  |  | K86.0 | | | Alcohol-induced chronic pancreatitis |
|  |  | O35.4 | | | Maternal care for (suspected) damage to foetus from alcohol |
|  |  | R78.0 | | | Finding of alcohol in blood |
|  |  | T51 | | | Toxic effect of alcohol |
|  |  | X45 | | | Accidental poisoning by and exposure to alcohol |
|  |  | Y15 | | | Poisoning by and exposure to alcohol, undetermined intent |
|  |  | Y90 | | | Evidence of alcohol involvement determined by blood alcohol level |
|  |  | Y91 | | | Evidence of alcohol involvement determined by level of intoxication |
|  |  | Z50.2 | | | Alcohol rehabilitation |
|  |  | Z71.4 | | | Alcohol abuse counselling and surveillance |
|  |  | Z72.1 | | | Problems related to lifestyle, alcohol use |
| Violence | | T74 | | | Maltreatment syndrome |
|  |  | T73 | | | Effects of other deprivation (extreme neglect) |
|  |  | Y06, Y07 | | | Perpetrator of neglect and other maltreatment syndromes |
|  |  | Y04, Y05 | | | Assault by bodily force and sexual assault |
|  |  | X85- Y03, Y08, Y09 | | | other type of assault |
|  |  | Y20-Y34 | | | Events of undetermined intent |
|  |  | Z04.5 | | | Examination and observation following other inflicted injury |
|  |  | Z04.8 | | | Examination and observation for other reasons: request for expert evidence |
| Self Harm | | X60-69 | | | Intentional self-poisoning |
|  |  | X70-X84 | | | Intentional self-harm |
|  |  | Z91.5 | | | Personal history of self-harm |

*Hospital admissions were created by linking finished consultant episodes into a continuous inpatient stay (including hospital transfers); **Diagnosis (in any position) accompanied by episode start date [epistart] within follow-up time and an emergency admission method [admimeth = 21-24, 28] in the first episode of the admission; ***In any diagnostic position accompanied by a primary diagnosis of injury (any ICD-10 code beginning with “S” or “T”, injuries, poisoning, and other consequences of external causes) and episode start date [epistart] within follow-up time and an emergency admission method [admimeth = 21-24, 28] in the first episode of the admission; ICD-10 = International Classification of Diseases 10th Revision.
Note: Mental disorders include mental health diagnoses as indicated by an F chapter (mental, behavioural, and neurodevelopmental disorders) ICD-10 code in any diagnostic position. Adversity-related injuries were indicated by any S or T (injuries, poisoning, and other consequences of external causes) chapter ICD-10 code alongside an ICD-10 code indicating that the harm was either self-inflicted or inflicted by another person (as opposed to accidental) in any diagnostic position.

**Table S5.** ICD-10 codes used to identify admissions with potentially psychosomatic symptoms in hospital episode statistics admitted patient care (including medical and surgical exclusion codes)

|  | ICD-10 codes | | ICD-10 Description |
| --- | --- | --- | --- |
| Potentially psychosomatic symptoms* ** |  | |  |
| Abdominal/pelvic pain | R10 | Abdominal and pelvic pain | |
| Headache | R51 | Headache | |
|  | G442 | Tension-type headache | |
| Other pain | M54 | Panniculitis affecting regions of neck and back | |
|  | M626 | Muscle strain | |
|  | M796 | Pain in limb | |
|  | R52 | Acute pain | |
| Circulatory/respiratory signs | R00 | Abnormalities of heart beat | |
|  | R03 | Abnormal blood pressure reading, without diagnosis | |
|  | R05 | Cough | |
|  | R06 | Abnormalities of breathing | |
|  | R07 | Pain in throat and chest | |
| Digestive symptoms | R11-14 | Nausea and vomiting, Heartburn, Dysphagia, Flatulence and related conditions | |
|  | R194 | Change in bowel habit | |
| Skin symptoms | R20-21 | Disturbances of skin sensation, rash and other nonspecific skin eruption | |
|  | R231 | Pallor | |
|  | R234 | Changes in skin texture | |
|  | R238 | Other and unspecified skin changes | |
| Nervous/musculoskeletal symptoms | R25 | Abnormal involuntary movements | |
|  | R26 | Abnormalities of gait and mobility | |
|  | R27 | Other lack of co-ordination | |
|  | R29.2-29.4 | Abnormal reflex, Abnormal posture, Clicking hip | |
|  | R298 | Other and unspecified signs and symptoms involving nervous and musculoskeletal systems | |
| Cognitive symptoms | R40.0-40.1 | Somnolence, Stupor | |
|  | R41-42 | Other symptoms and signs involving cognitive functions and awareness, Dizziness and giddiness | |
| Malaise/Fatigue/Syncope | R53 | Malaise and fatigue | |
|  | R55 | Syncope and collapse | |
| Other/general symptoms | R44 | Other symptoms and signs involving general sensations and perceptions | |
|  | R45 | Symptoms and signs involving emotional state | |
|  | R46 | Symptoms and signs involving appearance and behaviour | |
|  | R47 | Dysphasia and aphasia | |
|  | R49 | Voice disturbances | |
|  | Z56.3-56.4 | Stressful work schedule, Discord with boss and workmates | |
|  | Z71.1 | Person with feared complaint in whom no diagnosis is made | |
|  | Z73.3 | Stress, not elsewhere classified | |
| Sleep disorders | F51 | Nonorganic sleep disorders | |
|  | G47 | Disorders of initiating and maintaining sleep [insomnias] | |

| Medical and surgical exclusion codes |  |  |
| --- | --- | --- |
| Medical | A00-09 | Intestinal infectious diseases |
|  | K52.0 | Gastroenteritis and colitis due to radiation |
|  | K52.1 | Toxic gastroenteritis and colitis |
|  | K52.9 | Noninfective gastroenteritis and colitis unspecified |
|  | J09-10, J13-18 | Influenza and pneumonia |
|  | N81, N83, N85-90 | Non-inflammatory disease of female genital tract  Female genital prolapse |
|  | E28.2 | Polycystic ovarian syndrome |
|  | N31 | Neuromuscular dysfunction of bladder not elsewhere classified |
|  | N39.0 | Urinary tract infection site not specified |
|  | K55 | Vascular disorders of intestine |
|  | K56 | Paralytic ileus and intestinal obstruction without hernia |
|  | G40 | Epilepsy |
|  | G41 | Status epilepticus |
|  | G45 | Transient cerebral ischaemic attacks and related syndromes |
|  | G46 | Vascular syndromes of brain in cerebrovascular diseases |
|  | I60-69 | Cerebrovascular disease |
|  | C15-26 | Malignant neoplasms of digestive organs |
|  | C30-39 | Malignant neoplasms of respiratory and intrathoracic organs |
|  | C40-41 | Malignant neoplasms of bone and articular cartilage |
|  | C45-49 | Malignant neoplasms of mesothelial and soft tissue |
|  | C51-58 | Malignant neoplasms of female genital organs |
|  | C64-68 | Malignant neoplasms of urinary tract |
|  | C69-72 | Malignant neoplasms of eye brain and other parts of central nervous system |
|  | C73-75 | Malignant neoplasms of thyroid and other endocrine glands |
|  | K35-38 | Diseases of appendix |
| Surgical | Y75.2 | Laparoscopic approach to the abdominal cavity not elsewhere classified |
|  | H01.1 | Emergency excision of abnormal appendix and drainage HFQ |
|  | H01.2 | Emergency excision of normal appendix not elsewhere classified |
|  | H01.3 | Emergency excision of normal appendix |
|  | H01.9 | Unspecified emergency excision of appendix |
|  | H02.1 | Interval appendectomy |
|  | H02.3 | Prophylactic appendectomy NEC |
|  | H02.4 | Incidental appendectomy |
|  | H02.8 | Other specified excision of appendix |
|  | H02.9 | Unspecified excision of appendix |

*Diagnosis (in any diagnostic position) accompanied by episode start date [epistart] within follow-up time and an emergency admission method [admimeth = 21-24, 28] in the first episode of the admission; **Admissions were not categorised as potentially psychosomatic if a medical or surgical cause was indicated by an operation or subsidiary diagnostic code for the same admission; ICD-10 = International Classification of Diseases 10th Revision.
Note: ICD-10 codes related to pain, cardiovascular/respiratory, digestive, skin, sleep and fatigue are used to indicate potential manifestations of stress as the main reason for admission (when not accompanied by a medical or surgical operation/diagnostic code in the same admission).

**Figure S3.** Flow diagram of cohort derivation and cohort appearance in outcome datasets; *Enrolment defined by appearance in January census of the School Census or the alternative provision census or pupil referral unit census; MSOA = middle layer super output area

**Table S6.** Linkage between the national pupil database and NHS-England datasets, by cohort characteristics

|  |  | **Total** | **Linked** | **Not linked** |
| --- | --- | --- | --- | --- |
|  |  | **N** | N (%) | N (%) |
|  | Total | 5896443 | 5629732 (95.5) | 266711 (4.5) |
| Cohort (Year 7 entry) | 2012/13 | 534016 | 503359 (94.3) | 30657 (5.7) |
|  | 2013/14 | 530854 | 503410 (94.8) | 27444 (5.2) |
|  | 2014/15 | 552023 | 526665 (95.4) | 25358 (4.6) |
|  | 2015/16 | 570233 | 542045 (95.1) | 28188 (4.9) |
|  | 2016/17 | 582917 | 554167 (95.1) | 28750 (4.9) |
|  | 2017/18 | 594928 | 566595 (95.2) | 28333 (4.8) |
|  | 2018/19 | 614782 | 586178 (95.4) | 28604 (4.7) |
|  | 2019/20 | 638689 | 612429 (95.9) | 26260 (4.1) |
|  | 2020/21 | 633154 | 612140 (96.7) | 21014 (3.3) |
|  | 2021/22 | 644847 | 622744 (96.6) | 22103 (3.4) |
| Gender | Male | 3017539 | 2886328 (95.7) | 131211 (4.4) |
|  | Female | 2878904 | 2743404 (95.3) | 135500 (4.7) |
| Region of residence* | East Midlands | 513310 | 494000 (96.2) | 19310 (3.8) |
|  | East of England | 664309 | 633420 (95.4) | 30885 (4.7) |
|  | London | 879251 | 817260 (93.0) | 61990 (7.1) |
|  | North East | 279511 | 271515 (97.1) | 7995 (2.9) |
|  | North West | 808461 | 769030 (95.1) | 39435 (4.9) |
|  | South East | 933727 | 895180 (95.9) | 38550 (4.1) |
|  | South West | 553318 | 533180 (96.4) | 20135 (3.6) |
|  | West Midlands | 663059 | 634915 (95.8) | 28145 (4.2) |
|  | Yorkshire and the Humber | 601497 | 581230 (96.6) | 20265 (3.4) |
| IMD groups | 1 Most deprived 20% | 1480504 | 1409706 (95.2) | 70798 (4.8) |
|  | 2 | 1196718 | 1141204 (95.4) | 55514 (4.6) |
|  | 3 | 1079446 | 1031046 (95.5) | 48400 (4.5) |
|  | 4 | 1031517 | 987215 (95.7) | 44302 (4.3) |
|  | 5 Least deprived 20% | 1108258 | 1060561 (95.7) | 47697 (4.3) |
| FSM eligible | No | 4830203 | 4612972 (95.5) | 217231 (4.5) |
|  | Yes | 1066240 | 1016760 (95.4) | 49480 (4.6) |
| Racial-ethnic group (minor) | Bangladeshi | 99809 | 93045 (93.2) | 6764 (6.8) |
|  | Indian | 168835 | 159566 (94.5) | 9269 (5.5) |
|  | Asian (other) | 101602 | 97120 (95.6) | 4482 (4.4) |
|  | Pakistani | 252193 | 237382 (94.1) | 14811 (5.9) |
|  | Black African | 218696 | 203439 (93.0) | 15257 (7.0) |
|  | Black Caribbean | 72555 | 61104 (84.2) | 11451 (15.8) |
|  | Black (other) | 42063 | 37927 (90.2) | 4136 (9.8) |
|  | Chinese | 23060 | 21877 (94.9) | 1183 (5.1) |
|  | Mixed (other) | 118202 | 110031 (93.1) | 8171 (6.9) |
|  | Mixed Asian & White | 78253 | 74257 (94.9) | 3996 (5.1) |
|  | Mixed Black African & White | 44676 | 41964 (93.9) | 2712 (6.1) |
|  | Mixed Black Caribbean & White | 91105 | 84974 (93.3) | 6131 (6.7) |
|  | Other | 103977 | 98279 (94.5) | 5698 (5.5) |
|  | Refused | 43336 | 39469 (91.1) | 3867 (8.9) |
|  | White British | 4064670 | 3917148 (96.4) | 147522 (3.6) |
|  | White Irish | 17594 | 16621 (94.5) | 973 (5.5) |
|  | White Irish Traveller | 3484 | 3301 (94.8) | 183 (5.3) |
|  | White (other) | 330378 | 311401 (94.3) | 18977 (5.7) |
|  | White Romany Gypsy | 17847 | 17083 (95.7) | 764 (4.3) |
|  | Missing | 4108 | 3744 (91.1) | 364 (8.9) |
| Recorded SEND provision | None | 4831458 | 4610725 (95.4) | 220735 (4.6) |
|  | SEND support | 838764 | 802935 (95.7) | 35829 (4.3) |
|  | EHCP | 226221 | 216074 (95.5) | 10147 (4.5) |
| Persistent absences | No | 5332305 | 5097450 (95.6) | 234855 (4.4) |
|  | Yes | 420312 | 400470 (95.3) | 19842 (4.7) |
|  | Missing | 143826 | 131812 (91.7) | 12014 (8.4) |
| Care experienced** | No | 5837050 | 5573340 (96) | 263710 (5) |
|  | Yes | 59400 | 56400 (95) | 3000 (5) |

*Counts rounded to the nearest 5 as per statistical disclosure rules for sub-national numbers; **Counts are rounded to the nearest 10 and percentages to the nearest whole number as per statistical disclosure rules for data from the Children Looked After dataset; IMD = index of multiple deprivation; FSM = free school meal; SEND = special educational needs and disability

**Table S7.** First referral to mental health services (overall and crisis referrals), by social strata

|  |  | Referrals (all) | | Crisis referrals* | |
| --- | --- | --- | --- | --- | --- |
|  |  | N | N crisis (% of all referrals) | N | Rate per 1000 person-years (95% CI) |
|  | Total | 980648 | 17486 (1.8) | 57204 | 2.36 (2.34, 2.37) |
| Gender | Male | 405483 | 5832 (1.4) | 16592 | 1.33 (1.31, 1.35) |
|  | Female | 575165 | 11654 (2.0) | 40612 | 3.44 (3.41, 3.47) |
| Region of residence** | East Midlands | 83745 | 1865 (2.2) | 5970 | 2.81 (2.74, 2.88) |
|  | East of England | 113545 | 1905 (1.7) | 7385 | 2.70 (2.64, 2.77) |
|  | London | 120665 | 3375 (2.8) | 9365 | 2.66 (2.61, 2.72) |
|  | North East | 58975 | 1115 (1.9) | 3800 | 3.24 (3.14, 3.34) |
|  | North West | 141510 | 2915 (2.1) | 8715 | 2.63 (2.57, 2.68) |
|  | South East | 162470 | 2190 (1.3) | 8210 | 2.13 (2.08, 2.17) |
|  | South West | 89675 | 510 (0.6) | 1845 | 0.80 (0.76, 0.83) |
|  | West Midlands | 113140 | 1395 (1.2) | 5105 | 1.86 (1.81, 1.91) |
|  | Yorkshire and the Humber | 96930 | 2210 (2.3) | 6810 | 2.72 (2.65, 2.78) |
| IMD groups | 1 Most deprived 20% | 265864 | 5816 (2.2) | 17672 | 2.93 (2.89, 2.97) |
|  | 2 | 210512 | 3936 (1.9) | 12793 | 2.61 (2.56, 2.65) |
|  | 3 | 180981 | 2795 (1.5) | 9548 | 2.14 (2.10, 2.19) |
|  | 4 | 162635 | 2452 (1.5) | 8595 | 2.01 (1.97, 2.05) |
|  | 5 Least deprived 20% | 160656 | 2487 (1.5) | 8596 | 1.86 (1.82, 1.90) |
| FSM eligible | No | 723741 | 12766 (1.8) | 40956 | 2.03 (2.01, 2.05) |
|  | Yes | 256907 | 4720 (1.8) | 16248 | 3.97 (3.91, 4.03) |
| Racial-ethnic group | Bangladeshi | 9060 | 345 (3.8) | 822 | 2.04 (1.91, 2.19) |
|  | Indian | 10738 | 262 (2.4) | 655 | 0.98 (0.91, 1.06) |
|  | Asian (other) | 7674 | 256 (3.3) | 600 | 1.48 (1.37, 1.60) |
|  | Pakistani | 20098 | 486 (2.4) | 1245 | 1.23 (1.16, 1.30) |
|  | Black African | 15900 | 720 (4.5) | 1457 | 1.71 (1.62, 1.80) |
|  | Black Caribbean | 10690 | 309 (2.9) | 816 | 3.00 (2.80, 3.21) |
|  | Black (other) | 4556 | 159 (3.5) | 362 | 2.28 (2.05, 2.52) |
|  | Chinese | 1547 | 45 (2.9) | 92 | 1.05 (0.85, 1.28) |
|  | Mixed (other) | 18333 | 416 (2.3) | 1312 | 2.98 (2.82, 3.14) |
|  | Mixed Asian & White | 12190 | 221 (1.8) | 752 | 2.48 (2.31, 2.67) |
|  | Mixed Black African & White | 7347 | 174 (2.4) | 536 | 3.20 (2.94, 3.49) |
|  | Mixed Black Caribbean & White | 19836 | 422 (2.1) | 1398 | 3.90 (3.70, 4.11) |
|  | Other | 10059 | 259 (2.6) | 623 | 1.55 (1.43, 1.67) |
|  | Refused | 6219 | 125 (2.0) | 407 | 2.60 (2.36, 2.87) |
|  | White British | 784222 | 12349 (1.6) | 43589 | 2.54 (2.52, 2.56) |
|  | White Irish | 3111 | 53 (1.7) | 178 | 2.41 (2.08, 2.80) |
|  | White Irish Traveller | 848 | 12 (1.4) | 43 | 3.04 (2.25, 4.09) |
|  | White (other) | 35515 | 818 (2.3) | 2176 | 1.73 (1.66, 1.81) |
|  | White Romany Gypsy | 2268 | 47 (2.1) | 120 | 1.64 (1.37, 1.96) |
|  | Missing | 437 | 8 (1.8) | 21 | 1.33 (0.86, 2.03) |
| Chronic condition | No | 878951 | 16115 (1.8) | 51691 | 2.29 (2.27, 2.31) |
|  | Yes | 101697 | 1371 (1.3) | 5513 | 3.25 (3.16, 3.33) |
| Recorded SEND provision | None | 697496 | 13643 (2.0) | 42332 | 2.13 (2.11, 2.15) |
|  | SEN support | 211379 | 3219 (1.5) | 12039 | 3.40 (3.34, 3.46) |
|  | EHCP | 71773 | 624 (0.9) | 2833 | 3.22 (3.10, 3.34) |
| Persistent absences | No | 842904 | 15410 (1.8) | 49260 | 2.24 (2.22, 2.26) |
|  | Yes | 122795 | 1769 (1.4) | 6999 | 4.00 (3.90, 4.09) |
|  | Missing | 14949 | 307 (2.1) | 945 | 1.77 (1.66, 1.89) |
| Care experienced*** | No | 956270 | 17060 (2) | 55300 | 2.30 (2.28, 2.32) |
|  | Yes | 24370 | 430 (2) | 1910 | 7.88 (7.53, 8.24) |

*First crisis referral during follow up included in this definition, whereas column four (“N crisis (% of all referrals)”) counts of the number of first referrals that are considered crisis (see Table S2); **Counts rounded to the nearest 5 as per statistical disclosure rules for sub-national numbers; ***Counts are rounded to the nearest 10 and percentages to the nearest whole number as per statistical disclosure rules for data from the Children Looked After dataset; IMD = index of multiple deprivation; FSM = free school meal; SEND = special educational needs and disability

**Table S8.** Year-adjusted rates of first referral to mental health services per 1000 person-years (95% confidence intervals): derived from age group-specific Poisson regression models with separate models for each group of social strata

|  |  | **Age group** | | |
| --- | --- | --- | --- | --- |
|  |  | **11-<16** | **16-<18** | **18-<21** |
|  | *N* people | 5,628,357 | 2,769,042 | 1,710,950 |
|  | Overall | 50.7 (50.6, 50.8) | 42.0 (41.8, 42.2) | 23.6 (23.4, 23.8) |
| Gender | Male | 40.1 (40.0, 40.3) | 32.9 (32.7, 33.2) | 19.4 (19.2, 19.7) |
|  | Female | 62.0 (61.8, 62.2) | 52.3 (52.0, 52.6) | 28.3 (28.1, 28.6) |
| Region | East Midlands | 48.3 (47.9, 48.7) | 41.6 (40.9, 42.2) | 24.5 (23.9, 25.1) |
|  | East of England | 51.8 (51.4, 52.1) | 44.6 (44.0, 45.1) | 25.1 (24.6, 25.7) |
|  | London | 41.4 (41.1, 41.7) | 37.6 (37.1, 38.1) | 19.8 (19.4, 20.3) |
|  | North East | 68.3 (67.6, 68.9) | 49.4 (48.5, 50.4) | 23.5 (22.7, 24.2) |
|  | North West | 55.3 (55.0, 55.6) | 41.5 (41.0, 42.0) | 24.3 (23.8, 24.7) |
|  | South East | 53.4 (53.1, 53.7) | 44.3 (43.8, 44.8) | 23.2 (22.8, 23.6) |
|  | South West | 48.1 (47.8, 48.5) | 42.6 (41.9, 43.2) | 21.6 (21.1, 22.1) |
|  | West Midlands | 51.8 (51.4, 52.1) | 42.1 (41.5, 42.7) | 26.4 (25.8, 26.9) |
|  | Yorkshire and The Humber | 47.9 (47.6, 48.3) | 39.1 (38.5, 39.7) | 25.0 (24.4, 25.5) |
| IMD groups | 1.Most deprived | 55.5 (55.3, 55.8) | 45.4 (45.0, 45.8) | 27.7 (27.3, 28.1) |
|  | 2 | 54.4 (54.1, 54.7) | 44.1 (43.7, 44.6) | 25.3 (24.9, 25.7) |
|  | 3 | 51.2 (50.9, 51.5) | 42.6 (42.1, 43.0) | 23.1 (22.7, 23.4) |
|  | 4 | 47.4 (47.1, 47.6) | 40.0 (39.5, 40.4) | 21.7 (21.3, 22.1) |
|  | 5.Least deprived | 43.0 (42.8, 43.3) | 37.0 (36.6, 37.4) | 19.2 (18.8, 19.5) |
| FSM eligible | No | 44.1 (44.0, 44.2) | 38.0 (37.8, 38.2) | 21.0 (20.8, 21.2) |
|  | Yes | 85.2 (84.8, 85.6) | 64.7 (64.1, 65.3) | 37.6 (37.0, 38.1) |
| Racial-ethnic group (minor) | Bangladeshi | 24.3 (23.7, 24.9) | 26.8 (25.7, 28.0) | 17.0 (15.9, 18.1) |
|  | Indian | 17.1 (16.7, 17.5) | 18.2 (17.5, 19.0) | 12.3 (11.6, 13.0) |
|  | Asian (other) | 19.8 (19.3, 20.4) | 22.7 (21.6, 23.7) | 14.5 (13.5, 15.6) |
|  | Pakistani | 21.3 (20.9, 21.7) | 22.8 (22.1, 23.4) | 15.3 (14.6, 15.9) |
|  | Black African | 18.8 (18.4, 19.2) | 23.0 (22.3, 23.8) | 17.0 (16.2, 17.8) |
|  | Black Caribbean | 49.2 (48.1, 50.3) | 42.1 (40.4, 43.9) | 24.1 (22.6, 25.7) |
|  | Black (other) | 31.8 (30.7, 32.9) | 33.1 (31.0, 35.2) | 20.9 (18.8, 23.0) |
|  | Chinese | 18.3 (17.1, 19.4) | 21.3 (19.1, 23.5) | 13.7 (11.6, 15.8) |
|  | Mixed (other) | 49.5 (48.6, 50.3) | 45.8 (44.3, 47.4) | 25.4 (24.0, 26.9) |
|  | Mixed Asian & White | 47.6 (46.7, 48.6) | 45.1 (43.2, 46.9) | 24.3 (22.6, 26.0) |
|  | Mixed Black African & White | 52.1 (50.7, 53.4) | 46.8 (44.2, 49.4) | 28.8 (26.2, 31.4) |
|  | Mixed Black Caribbean & White | 71.8 (70.7, 72.9) | 57.7 (55.7, 59.6) | 34.8 (33.0, 36.7) |
|  | Other | 27.4 (26.8, 28.0) | 29.6 (28.4, 30.9) | 16.1 (15.0, 17.3) |
|  | Refused | 46.5 (45.2, 47.8) | 43.9 (41.3, 46.6) | 23.2 (20.8, 25.6) |
|  | White British | 59.7 (59.6, 59.9) | 46.8 (46.5, 47.0) | 25.5 (25.3, 25.7) |
|  | White Irish | 53.2 (51.0, 55.4) | 44.6 (41.1, 48.1) | 27.5 (24.2, 30.8) |
|  | White Irish Traveller | 70.4 (64.6, 76.2) | 74.2 (63.3, 85.2) | 65.3 (52.9, 77.8) |
|  | White (other) | 32.0 (31.6, 32.4) | 30.9 (30.2, 31.7) | 17.9 (17.2, 18.6) |
|  | White Romany Gypsy | 35.8 (34.1, 37.6) | 35.2 (32.0, 38.4) | 20.2 (17.3, 23.2) |
|  | Missing | 31.2 (27.6, 34.8) | 31.4 (25.2, 37.6) | 20.7 (14.8, 26.6) |

FSM = free school meals; IMD = index of multiple deprivation

**Table S9.** Year-adjusted incidence rate ratios of first referral to mental health services (95% confidence intervals), compared to the age- and social strata-specific mean rate: derived from age group-specific Poisson regression models with separate models for each group of social strata

|  |  | **Age group** | | |
| --- | --- | --- | --- | --- |
|  |  | **11-<16** | **16-<18** | **18-<21** |
|  | *N* people | 5,628,357 | 2,769,042 | 1,710,950 |
| Gender | Male | 0.81 (0.81, 0.81) | 0.80 (0.80, 0.81) | 0.84 (0.83, 0.84) |
|  | Female | 1.25 (1.25, 1.25) | 1.28 (1.27, 1.28) | 1.22 (1.21, 1.23) |
| Region | East Midlands | 0.96 (0.95, 0.97) | 0.99 (0.98, 1.01) | 1.04 (1.02, 1.07) |
|  | East of England | 1.03 (1.02, 1.03) | 1.06 (1.05, 1.08) | 1.07 (1.05, 1.09) |
|  | London | 0.82 (0.82, 0.83) | 0.90 (0.89, 0.91) | 0.84 (0.83, 0.86) |
|  | North East | 1.35 (1.34, 1.37) | 1.18 (1.16, 1.20) | 1.00 (0.97, 1.03) |
|  | North West | 1.10 (1.09, 1.10) | 0.99 (0.98, 1.00) | 1.03 (1.01, 1.05) |
|  | South East | 1.06 (1.05, 1.07) | 1.06 (1.05, 1.07) | 0.99 (0.97, 1.00) |
|  | South West | 0.96 (0.95, 0.96) | 1.02 (1.00, 1.03) | 0.92 (0.90, 0.94) |
|  | West Midlands | 1.03 (1.02, 1.03) | 1.00 (0.99, 1.02) | 1.12 (1.10, 1.14) |
|  | Yorkshire and The Humber | 0.95 (0.94, 0.96) | 0.93 (0.92, 0.95) | 1.06 (1.04, 1.08) |
| IMD groups | 1 Most deprived | 1.10 (1.10, 1.10) | 1.08 (1.08, 1.09) | 1.19 (1.17, 1.20) |
|  | 2 | 1.08 (1.07, 1.08) | 1.05 (1.04, 1.06) | 1.08 (1.07, 1.10) |
|  | 3 | 1.01 (1.01, 1.02) | 1.02 (1.01, 1.03) | 0.99 (0.97, 1.00) |
|  | 4 | 0.94 (0.93, 0.94) | 0.95 (0.95, 0.96) | 0.93 (0.91, 0.94) |
|  | 5 Least deprived | 0.85 (0.85, 0.86) | 0.88 (0.87, 0.89) | 0.82 (0.81, 0.83) |
| FSM eligible | No | 0.90 (0.89, 0.90) | 0.92 (0.92, 0.93) | 0.91 (0.91, 0.92) |
|  | Yes | 1.73 (1.72, 1.74) | 1.57 (1.56, 1.59) | 1.64 (1.61, 1.66) |
| Racial-ethnic group (minor) | Bangladeshi | 0.51 (0.50, 0.53) | 0.66 (0.63, 0.69) | 0.73 (0.69, 0.78) |
|  | Indian | 0.36 (0.35, 0.37) | 0.45 (0.43, 0.47) | 0.53 (0.50, 0.56) |
|  | Asian (other) | 0.42 (0.41, 0.43) | 0.56 (0.53, 0.58) | 0.63 (0.59, 0.67) |
|  | Pakistani | 0.45 (0.44, 0.46) | 0.56 (0.54, 0.58) | 0.66 (0.63, 0.69) |
|  | Black African | 0.40 (0.39, 0.40) | 0.57 (0.55, 0.58) | 0.73 (0.70, 0.77) |
|  | Black Caribbean | 1.04 (1.02, 1.06) | 1.04 (0.99, 1.08) | 1.04 (0.98, 1.11) |
|  | Black (other) | 0.67 (0.65, 0.70) | 0.81 (0.76, 0.87) | 0.90 (0.82, 1.00) |
|  | Chinese | 0.39 (0.36, 0.41) | 0.52 (0.47, 0.58) | 0.59 (0.51, 0.69) |
|  | Mixed (other) | 1.04 (1.03, 1.06) | 1.13 (1.09, 1.17) | 1.10 (1.04, 1.16) |
|  | Mixed Asian & White | 1.01 (0.99, 1.03) | 1.11 (1.06, 1.16) | 1.05 (0.98, 1.13) |
|  | Mixed Black African & White | 1.10 (1.07, 1.13) | 1.15 (1.09, 1.22) | 1.24 (1.14, 1.36) |
|  | Mixed Black Caribbean & White | 1.52 (1.49, 1.54) | 1.42 (1.37, 1.47) | 1.51 (1.43, 1.59) |
|  | Other | 0.58 (0.56, 0.59) | 0.73 (0.70, 0.76) | 0.70 (0.65, 0.75) |
|  | Refused | 0.98 (0.95, 1.01) | 1.08 (1.02, 1.15) | 1.00 (0.91, 1.11) |
|  | White British | 1.26 (1.26, 1.26) | 1.15 (1.15, 1.15) | 1.10 (1.10, 1.11) |
|  | White Irish | 1.12 (1.08, 1.17) | 1.10 (1.01, 1.19) | 1.19 (1.06, 1.34) |
|  | White Irish Traveller | 1.49 (1.37, 1.61) | 1.83 (1.58, 2.12) | 2.83 (2.34, 3.42) |
|  | White (other) | 0.68 (0.67, 0.68) | 0.76 (0.74, 0.78) | 0.78 (0.75, 0.81) |
|  | White Romany Gypsy | 0.76 (0.72, 0.79) | 0.87 (0.79, 0.95) | 0.88 (0.76, 1.01) |
|  | Missing | 0.66 (0.59, 0.74) | 0.77 (0.63, 0.94) | 0.90 (0.67, 1.19) |

FSM = free school meals; IMD = index of multiple deprivation

**Figure S4.** Year-adjusted incidence rate ratios (compared to the age- and social strata-specific mean rate) of first referral to mental health services with 95% confidence intervals: derived from age group-specific Poisson regression models with separate models for each group of social strata. FSM = free school meals; IMD = index of multiple deprivation.

**Figure S5.** Year-adjusted rates of first referral to mental health services per 1000 person-years: derived from separate Poisson regression models for each group of social strata and gender (11<16 years age group). FSM = free school meals; IMD = index of multiple deprivation.

**Figure S6.** Year-adjusted incidence rate ratios of first referral to mental health services (compared to the gender- and social strata-specific mean rate): derived from separate Poisson regression models for each group of social strata and gender (11<16 years age group). FSM = free school meals; IMD = index of multiple deprivation.

Emergency department attendances

**Table S10.** Year-adjusted rates of first mental health-related emergency department attendances (95% confidence intervals): derived from age group-specific Poisson regression models with separate models for each group of social strata

|  |  | **Age group** | | |
| --- | --- | --- | --- | --- |
|  |  | **11-<16** | **16-<18** | **18-<21** |
|  | *N* people | 3,553,224 | 2,167,870 | 2,055,710 |
|  | Overall | 11.2 (11.2, 11.3) | 15.9 (15.7, 16.1) | 17.4 (17.2, 17.5) |
| Gender | Male | 5.1 (5.0, 5.2) | 9.9 (9.7, 10.1) | 12.8 (12.6, 13.0) |
|  | Female | 17.7 (17.5, 17.9) | 22.3 (22.0, 22.6) | 22.3 (22.1, 22.6) |
| Region | East Midlands | 12.0 (11.7, 12.3) | 17.7 (17.1, 18.3) | 18.6 (18.1, 19.2) |
|  | East of England | 10.4 (10.2, 10.7) | 15.0 (14.5, 15.5) | 16.8 (16.4, 17.3) |
|  | London | 9.1 (8.9, 9.3) | 13.5 (13.1, 13.9) | 14.6 (14.3, 15.0) |
|  | North East | 12.4 (11.9, 12.8) | 17.7 (16.9, 18.5) | 19.3 (18.5, 20.0) |
|  | North West | 13.6 (13.3, 13.9) | 16.7 (16.3, 17.2) | 18.7 (18.3, 19.2) |
|  | South East | 11.2 (10.9, 11.4) | 16.3 (15.9, 16.8) | 17.3 (16.9, 17.7) |
|  | South West | 10.3 (10.0, 10.6) | 16.1 (15.5, 16.6) | 17.2 (16.7, 17.7) |
|  | West Midlands | 11.7 (11.4, 12.0) | 15.7 (15.2, 16.2) | 17.7 (17.2, 18.1) |
|  | Yorkshire and The Humber | 11.4 (11.1, 11.7) | 16.4 (15.8, 16.9) | 18.0 (17.5, 18.5) |
| IMD groups | 1.Most deprived | 12.7 (12.5, 12.9) | 17.9 (17.5, 18.2) | 19.3 (19.0, 19.6) |
|  | 2 | 12.5 (12.3, 12.7) | 17.4 (17.0, 17.8) | 18.9 (18.6, 19.3) |
|  | 3 | 11.0 (10.8, 11.2) | 16.1 (15.7, 16.5) | 17.3 (16.9, 17.6) |
|  | 4 | 10.1 (9.9, 10.3) | 14.3 (13.9, 14.6) | 16.1 (15.7, 16.5) |
|  | 5.Least deprived | 9.2 (9.0, 9.3) | 13.1 (12.8, 13.5) | 14.7 (14.4, 15.0) |
| FSM eligible | No | 9.9 (9.8, 10.0) | 14.1 (13.9, 14.3) | 15.7 (15.6, 15.9) |
|  | Yes | 16.9 (16.7, 17.2) | 26.1 (25.5, 26.6) | 25.5 (25.0, 25.9) |
| Racial-ethnic group (minor) | Bangladeshi | 6.4 (5.9, 6.9) | 9.2 (8.2, 10.2) | 9.7 (8.8, 10.6) |
|  | Indian | 4.2 (3.9, 4.5) | 6.9 (6.2, 7.6) | 9.1 (8.4, 9.8) |
|  | Asian (other) | 5.5 (5.0, 6.0) | 8.6 (7.6, 9.6) | 11.0 (10.0, 12.0) |
|  | Pakistani | 5.4 (5.1, 5.7) | 8.1 (7.5, 8.6) | 9.6 (9.0, 10.1) |
|  | Black African | 5.9 (5.6, 6.3) | 10.1 (9.4, 10.8) | 13.1 (12.4, 13.9) |
|  | Black Caribbean | 10.7 (9.8, 11.5) | 14.3 (12.8, 15.8) | 16.9 (15.5, 18.3) |
|  | Black (other) | 8.8 (7.9, 9.8) | 12.6 (10.8, 14.4) | 16.3 (14.3, 18.2) |
|  | Chinese | 4.1 (3.3, 4.9) | 8.9 (6.8, 11.1) | 8.2 (6.4, 10.0) |
|  | Mixed (other) | 11.4 (10.8, 12.0) | 17.9 (16.6, 19.3) | 19.2 (17.9, 20.5) |
|  | Mixed Asian & White | 11.4 (10.7, 12.2) | 16.5 (15.0, 18.1) | 19.2 (17.6, 20.8) |
|  | Mixed Black African & White | 14.0 (12.9, 15.1) | 19.5 (17.2, 21.7) | 22.7 (20.3, 25.1) |
|  | Mixed Black Caribbean & White | 16.3 (15.4, 17.2) | 23.9 (22.1, 25.6) | 23.3 (21.8, 24.8) |
|  | Other | 7.2 (6.7, 7.7) | 10.5 (9.5, 11.6) | 11.7 (10.7, 12.8) |
|  | Refused | 10.6 (9.7, 11.6) | 16.9 (14.7, 19.1) | 17.9 (15.7, 20.1) |
|  | White British | 12.8 (12.7, 13.0) | 17.6 (17.4, 17.8) | 18.8 (18.6, 19.0) |
|  | White Irish | 13.0 (11.1, 14.8) | 17.3 (14.2, 20.5) | 16.8 (14.1, 19.5) |
|  | White Irish Traveller | 11.5 (7.7, 15.2) | 23.5 (14.8, 32.2) | 29.3 (21.0, 37.5) |
|  | White (other) | 8.4 (8.1, 8.7) | 12.0 (11.4, 12.7) | 13.5 (12.8, 14.1) |
|  | White Romany Gypsy | 7.9 (6.6, 9.3) | 14.3 (11.5, 17.2) | 12.8 (10.3, 15.3) |
|  | Missing | 8.2 (5.1, 11.2) | 5.2 (1.3, 9.1) | 18.6 (12.5, 24.6) |

FSM = free school meals; IMD = index of multiple deprivation

**Table S11.** Year-adjusted incidence rate ratios of first mental health-related emergency department attendance (95% confidence intervals), compared to the age- and social strata-specific mean rate: derived from age group-specific Poisson regression models with separate models for each group of social strata

|  |  | **Age group** | | |
| --- | --- | --- | --- | --- |
|  |  | **11-<16** | **16-<18** | **18-<21** |
| Gender | Male | 0.55 (0.54, 0.55) | 0.66 (0.65, 0.66) | 0.75 (0.74, 0.75) |
|  | Female | 1.89 (1.87, 1.91) | 1.56 (1.54, 1.59) | 1.37 (1.35, 1.38) |
| Region | East Midlands | 1.08 (1.05, 1.10) | 1.13 (1.09, 1.17) | 1.08 (1.05, 1.11) |
|  | East of England | 0.93 (0.91, 0.96) | 0.94 (0.91, 0.97) | 0.97 (0.94, 1.00) |
|  | London | 0.81 (0.80, 0.83) | 0.84 (0.81, 0.86) | 0.83 (0.81, 0.85) |
|  | North East | 1.11 (1.07, 1.14) | 1.13 (1.08, 1.19) | 1.12 (1.08, 1.17) |
|  | North West | 1.22 (1.20, 1.24) | 1.06 (1.03, 1.09) | 1.09 (1.06, 1.11) |
|  | South East | 1.00 (0.98, 1.02) | 1.03 (1.01, 1.06) | 1.00 (0.98, 1.02) |
|  | South West | 0.92 (0.90, 0.94) | 1.01 (0.98, 1.05) | 0.99 (0.96, 1.02) |
|  | West Midlands | 1.05 (1.02, 1.07) | 0.99 (0.96, 1.02) | 1.02 (0.99, 1.05) |
|  | Yorkshire and The Humber | 1.02 (1.00, 1.05) | 1.03 (1.00, 1.07) | 1.04 (1.01, 1.07) |
| IMD groups | 1.Most deprived | 1.14 (1.12, 1.15) | 1.14 (1.12, 1.16) | 1.12 (1.11, 1.14) |
|  | 2 | 1.12 (1.10, 1.14) | 1.11 (1.08, 1.13) | 1.10 (1.08, 1.12) |
|  | 3 | 0.99 (0.97, 1.01) | 1.02 (0.99, 1.04) | 1.00 (0.98, 1.02) |
|  | 4 | 0.91 (0.89, 0.92) | 0.89 (0.87, 0.92) | 0.93 (0.91, 0.95) |
|  | 5.Least deprived | 0.82 (0.80, 0.83) | 0.82 (0.80, 0.84) | 0.84 (0.82, 0.86) |
| FSM eligible | No | 0.90 (0.90, 0.91) | 0.90 (0.90, 0.91) | 0.92 (0.91, 0.92) |
|  | Yes | 1.54 (1.52, 1.56) | 1.76 (1.71, 1.80) | 1.54 (1.51, 1.57) |
| Racial-ethnic group (minor) | Bangladeshi | 0.59 (0.55, 0.64) | 0.58 (0.52, 0.64) | 0.55 (0.50, 0.61) |
|  | Indian | 0.39 (0.36, 0.42) | 0.43 (0.39, 0.47) | 0.52 (0.48, 0.56) |
|  | Asian (other) | 0.51 (0.47, 0.56) | 0.54 (0.48, 0.60) | 0.63 (0.57, 0.69) |
|  | Pakistani | 0.50 (0.48, 0.53) | 0.50 (0.47, 0.54) | 0.54 (0.51, 0.58) |
|  | Black African | 0.55 (0.52, 0.58) | 0.63 (0.59, 0.68) | 0.75 (0.71, 0.80) |
|  | Black Caribbean | 0.99 (0.91, 1.08) | 0.92 (0.82, 1.03) | 0.99 (0.91, 1.08) |
|  | Black (other) | 0.82 (0.74, 0.91) | 0.80 (0.69, 0.94) | 0.95 (0.84, 1.08) |
|  | Chinese | 0.38 (0.31, 0.47) | 0.56 (0.43, 0.72) | 0.46 (0.37, 0.58) |
|  | Mixed (other) | 1.06 (1.00, 1.12) | 1.17 (1.08, 1.27) | 1.14 (1.06, 1.23) |
|  | Mixed Asian & White | 1.06 (1.00, 1.14) | 1.07 (0.97, 1.19) | 1.14 (1.04, 1.25) |
|  | Mixed Black African & White | 1.30 (1.21, 1.41) | 1.28 (1.12, 1.45) | 1.36 (1.21, 1.53) |
|  | Mixed Black Caribbean & White | 1.52 (1.44, 1.61) | 1.62 (1.49, 1.75) | 1.40 (1.31, 1.51) |
|  | Other | 0.67 (0.62, 0.72) | 0.67 (0.60, 0.74) | 0.67 (0.61, 0.74) |
|  | Refused | 0.99 (0.90, 1.08) | 1.10 (0.95, 1.26) | 1.05 (0.92, 1.20) |
|  | White British | 1.20 (1.19, 1.20) | 1.15 (1.14, 1.16) | 1.11 (1.11, 1.12) |
|  | White Irish | 1.21 (1.05, 1.39) | 1.13 (0.93, 1.38) | 0.99 (0.83, 1.18) |
|  | White Irish Traveller | 1.07 (0.77, 1.49) | 1.61 (1.06, 2.44) | 1.80 (1.31, 2.46) |
|  | White (other) | 0.78 (0.75, 0.81) | 0.77 (0.72, 0.81) | 0.78 (0.74, 0.82) |
|  | White Romany Gypsy | 0.74 (0.62, 0.87) | 0.93 (0.75, 1.15) | 0.73 (0.60, 0.90) |
|  | Missing | 0.76 (0.52, 1.10) | 0.32 (0.15, 0.69) | 1.09 (0.77, 1.55) |

FSM = free school meals; IMD = index of multiple deprivation

**Figure Figure S7.** Year-adjusted incidence rate ratios (compared to the age- and social strata-specific mean rate) of first mental health-related emergency department attendance with 95% confidence intervals: derived from age group-specific Poisson regression models with separate models for each group of social strata. FSM = free school meals; IMD = index of multiple deprivation

**Figure S8.** Year-adjusted rates of first mental health-related emergency department attendance per 1000 person-years: derived from separate Poisson regression models for each group of social strata and gender (11<16 years age group). FSM = free school meals; IMD = index of multiple deprivation

**Figure S9.** Year-adjusted incidence rate ratios of referrals to mental health services (compared to the gender- and social strata-specific mean rate): derived from separate Poisson regression models for each group of social strata and gender (11<16 years group). FSM = free school meals; IMD = index of multiple deprivation

Mental health-related emergency hospital admissions

**Table S12.** Year-adjusted rates of first mental health-related emergency hospital admission (95% confidence intervals): derived from age group-specific Poisson regression models with separate models for each group of social strata

|  |  | **Age group** | | |
| --- | --- | --- | --- | --- |
|  |  | **11-<16** | **16-<18** | **18-<21** |
|  | *N* people | 5,628,357 | 2,769,042 | 1,710,950 |
|  | Overall | 3.8 (3.8, 3.9) | 4.9 (4.8, 5.0) | 3.9 (3.8, 3.9) |
| Gender | Male | 1.4 (1.4, 1.4) | 2.7 (2.6, 2.8) | 2.7 (2.6, 2.8) |
|  | Female | 6.4 (6.3, 6.4) | 7.3 (7.2, 7.4) | 5.2 (5.0, 5.3) |
| Region | East Midlands | 3.3 (3.2, 3.4) | 4.6 (4.4, 4.8) | 4.2 (4.0, 4.4) |
|  | East of England | 3.3 (3.2, 3.3) | 3.9 (3.7, 4.1) | 3.5 (3.3, 3.7) |
|  | London | 2.4 (2.4, 2.5) | 3.4 (3.3, 3.5) | 2.7 (2.6, 2.9) |
|  | North East | 4.6 (4.5, 4.8) | 5.9 (5.6, 6.2) | 5.1 (4.8, 5.5) |
|  | North West | 5.2 (5.1, 5.3) | 4.7 (4.5, 4.8) | 4.4 (4.2, 4.5) |
|  | South East | 3.8 (3.7, 3.8) | 6.3 (6.1, 6.5) | 4.5 (4.3, 4.7) |
|  | South West | 5.0 (4.9, 5.1) | 7.6 (7.3, 7.8) | 4.6 (4.4, 4.8) |
|  | West Midlands | 4.2 (4.1, 4.2) | 4.4 (4.2, 4.6) | 3.4 (3.2, 3.6) |
|  | Yorkshire and The Humber | 3.4 (3.3, 3.4) | 4.2 (4.0, 4.4) | 3.3 (3.1, 3.4) |
| IMD groups | 1.Most deprived | 4.2 (4.2, 4.3) | 5.2 (5.0, 5.3) | 4.4 (4.2, 4.5) |
|  | 2 | 4.1 (4.0, 4.1) | 5.2 (5.0, 5.3) | 4.2 (4.1, 4.4) |
|  | 3 | 3.9 (3.8, 3.9) | 5.1 (4.9, 5.2) | 3.7 (3.5, 3.8) |
|  | 4 | 3.6 (3.5, 3.6) | 4.6 (4.5, 4.7) | 3.6 (3.4, 3.7) |
|  | 5.Least deprived | 3.2 (3.2, 3.3) | 4.4 (4.3, 4.5) | 3.4 (3.2, 3.5) |
| FSM eligible | No | 3.3 (3.3, 3.4) | 4.4 (4.3, 4.4) | 3.5 (3.4, 3.5) |
|  | Yes | 6.3 (6.2, 6.4) | 7.7 (7.5, 7.9) | 5.9 (5.7, 6.0) |
| Racial-ethnic group (minor) | Bangladeshi | 2.0 (1.8, 2.1) | 2.5 (2.1, 2.8) | 2.5 (2.1, 2.9) |
|  | Indian | 1.2 (1.1, 1.3) | 1.9 (1.7, 2.2) | 1.7 (1.4, 1.9) |
|  | Asian (other) | 1.5 (1.4, 1.7) | 2.6 (2.2, 2.9) | 2.7 (2.3, 3.2) |
|  | Pakistani | 1.5 (1.5, 1.6) | 2.3 (2.1, 2.5) | 2.1 (1.9, 2.4) |
|  | Black African | 1.4 (1.3, 1.5) | 2.4 (2.1, 2.6) | 2.6 (2.3, 2.9) |
|  | Black Caribbean | 2.9 (2.7, 3.1) | 3.2 (2.8, 3.7) | 3.2 (2.7, 3.7) |
|  | Black (other) | 2.3 (2.0, 2.5) | 3.4 (2.7, 4.0) | 3.9 (3.1, 4.7) |
|  | Chinese | 1.3 (1.1, 1.6) | 2.3 (1.6, 3.0) | 1.9 (1.1, 2.6) |
|  | Mixed (other) | 4.0 (3.8, 4.2) | 5.4 (4.9, 5.9) | 4.2 (3.6, 4.7) |
|  | Mixed Asian & White | 4.1 (3.9, 4.4) | 5.5 (4.9, 6.1) | 4.5 (3.9, 5.2) |
|  | Mixed Black African & White | 4.2 (3.9, 4.6) | 5.4 (4.6, 6.2) | 4.4 (3.4, 5.3) |
|  | Mixed Black Caribbean & White | 5.3 (5.1, 5.6) | 6.6 (6.0, 7.2) | 4.7 (4.1, 5.3) |
|  | Other | 2.0 (1.9, 2.2) | 2.5 (2.2, 2.9) | 2.8 (2.4, 3.3) |
|  | Refused | 3.8 (3.5, 4.1) | 5.6 (4.7, 6.5) | 3.7 (2.8, 4.5) |
|  | White British | 4.4 (4.4, 4.5) | 5.5 (5.4, 5.6) | 4.2 (4.2, 4.3) |
|  | White Irish | 4.1 (3.6, 4.6) | 5.6 (4.4, 6.7) | 3.5 (2.4, 4.5) |
|  | White Irish Traveller | 5.0 (3.7, 6.2) | 4.7 (2.2, 7.2) | 7.4 (3.8, 11.0) |
|  | White (other) | 2.7 (2.6, 2.8) | 3.5 (3.2, 3.7) | 2.7 (2.4, 2.9) |
|  | White Romany Gypsy | 2.5 (2.1, 2.9) | 5.0 (3.9, 6.1) | 3.2 (2.1, 4.3) |
|  | Missing | 2.7 (1.8, 3.6) | 5.5 (3.0, 8.0) | 2.6 (0.7, 4.5) |

FSM = free school meals; IMD = index of multiple deprivation

**Table S13.** Year-adjusted incidence rate ratios of first mental health-related emergency hospital admission (95% confidence intervals), compared to the age- and social strata-specific mean rate: derived from age group-specific Poisson regression models with separate models for each group of social strata

|  |  | **Age group** | | |
| --- | --- | --- | --- | --- |
|  |  | **11-<16** | **16-<18** | **18-<21** |
|  | *N* people | 5,628,357 | 2,769,042 | 1,710,950 |
| Gender | Male | 0.48 (0.47, 0.48) | 0.62 (0.61, 0.63) | 0.74 (0.73, 0.75) |
|  | Female | 2.18 (2.16, 2.20) | 1.68 (1.66, 1.71) | 1.40 (1.37, 1.42) |
| Region | East Midlands | 0.89 (0.87, 0.91) | 0.96 (0.93, 1.01) | 1.11 (1.05, 1.17) |
|  | East of England | 0.88 (0.86, 0.89) | 0.82 (0.79, 0.85) | 0.92 (0.87, 0.96) |
|  | London | 0.66 (0.64, 0.67) | 0.72 (0.69, 0.74) | 0.72 (0.69, 0.75) |
|  | North East | 1.24 (1.21, 1.28) | 1.23 (1.17, 1.30) | 1.35 (1.26, 1.43) |
|  | North West | 1.39 (1.37, 1.41) | 0.98 (0.95, 1.01) | 1.14 (1.10, 1.19) |
|  | South East | 1.01 (1.00, 1.03) | 1.33 (1.29, 1.36) | 1.18 (1.14, 1.22) |
|  | South West | 1.33 (1.31, 1.36) | 1.59 (1.54, 1.64) | 1.21 (1.16, 1.27) |
|  | West Midlands | 1.12 (1.10, 1.14) | 0.93 (0.89, 0.96) | 0.89 (0.85, 0.94) |
|  | Yorkshire and The Humber | 0.90 (0.89, 0.92) | 0.88 (0.84, 0.91) | 0.85 (0.81, 0.90) |
| IMD groups | 1.Most deprived | 1.11 (1.10, 1.13) | 1.06 (1.03, 1.08) | 1.13 (1.10, 1.16) |
|  | 2 | 1.07 (1.06, 1.09) | 1.05 (1.03, 1.08) | 1.10 (1.07, 1.14) |
|  | 3 | 1.02 (1.00, 1.03) | 1.04 (1.01, 1.07) | 0.96 (0.92, 0.99) |
|  | 4 | 0.93 (0.92, 0.95) | 0.94 (0.91, 0.97) | 0.93 (0.89, 0.96) |
|  | 5.Least deprived | 0.84 (0.83, 0.86) | 0.90 (0.88, 0.93) | 0.87 (0.84, 0.90) |
| FSM eligible | No | 0.90 (0.89, 0.90) | 0.91 (0.91, 0.92) | 0.92 (0.91, 0.92) |
|  | Yes | 1.69 (1.67, 1.71) | 1.61 (1.57, 1.64) | 1.54 (1.50, 1.59) |
| Racial-ethnic group (minor) | Bangladeshi | 0.55 (0.51, 0.59) | 0.52 (0.46, 0.60) | 0.66 (0.56, 0.77) |
|  | Indian | 0.34 (0.32, 0.37) | 0.41 (0.37, 0.46) | 0.44 (0.38, 0.51) |
|  | Asian (other) | 0.43 (0.39, 0.46) | 0.55 (0.48, 0.63) | 0.72 (0.62, 0.84) |
|  | Pakistani | 0.43 (0.41, 0.45) | 0.49 (0.45, 0.53) | 0.56 (0.51, 0.63) |
|  | Black African | 0.40 (0.38, 0.42) | 0.50 (0.46, 0.55) | 0.68 (0.61, 0.76) |
|  | Black Caribbean | 0.81 (0.75, 0.88) | 0.68 (0.60, 0.79) | 0.85 (0.72, 0.99) |
|  | Black (other) | 0.63 (0.57, 0.71) | 0.72 (0.60, 0.87) | 1.03 (0.83, 1.28) |
|  | Chinese | 0.37 (0.31, 0.45) | 0.49 (0.36, 0.67) | 0.50 (0.33, 0.74) |
|  | Mixed (other) | 1.11 (1.05, 1.16) | 1.15 (1.05, 1.26) | 1.11 (0.97, 1.25) |
|  | Mixed Asian & White | 1.15 (1.08, 1.21) | 1.16 (1.04, 1.30) | 1.20 (1.03, 1.39) |
|  | Mixed Black African & White | 1.18 (1.09, 1.27) | 1.16 (0.99, 1.35) | 1.16 (0.94, 1.43) |
|  | Mixed Black Caribbean & White | 1.48 (1.41, 1.55) | 1.40 (1.28, 1.53) | 1.23 (1.09, 1.40) |
|  | Other | 0.56 (0.52, 0.60) | 0.54 (0.47, 0.62) | 0.75 (0.64, 0.88) |
|  | Refused | 1.06 (0.97, 1.15) | 1.20 (1.02, 1.40) | 0.97 (0.76, 1.23) |
|  | White British | 1.23 (1.22, 1.24) | 1.18 (1.17, 1.19) | 1.12 (1.11, 1.14) |
|  | White Irish | 1.13 (1.00, 1.28) | 1.18 (0.96, 1.46) | 0.91 (0.67, 1.24) |
|  | White Irish Traveller | 1.38 (1.07, 1.78) | 1.01 (0.60, 1.70) | 1.95 (1.20, 3.18) |
|  | White (other) | 0.75 (0.73, 0.78) | 0.74 (0.69, 0.79) | 0.71 (0.65, 0.78) |
|  | White Romany Gypsy | 0.70 (0.60, 0.82) | 1.06 (0.85, 1.33) | 0.85 (0.60, 1.20) |
|  | Missing | 0.76 (0.55, 1.05) | 1.17 (0.75, 1.84) | 0.69 (0.33, 1.44) |

FSM = free school meals; IMD = index of multiple deprivation

**Figure S10.** Year-adjusted incidence rate ratios (compared to the age- and social strata-specific mean rate) of first mental health-related emergency hospital admission with 95% confidence intervals: derived from age group-specific Poisson regression models with separate models for each group of social strata. FSM = free school meals; IMD = index of multiple deprivation

**Figure S11.** Year-adjusted rates of first mental health-related emergency hospital admission per 1000 person-years: derived from separate Poisson regression models for each group of social strata and gender (11<16 years group). FSM = free school meals; IMD = index of multiple deprivation

**Figure S12.** Year-adjusted incidence rate ratios of first mental health-related emergency hospital admission (compared to the gender- and social strata-specific mean rate): derived from separate Poisson regression models for each group of social strata and gender (11-<16 years group). FSM = free school meals; IMD = index of multiple deprivation

Mental health-related emergency hospital admissions (including potentially psychosomatic symptoms)

**Table S14.** Year-adjusted rates of first mental health-related emergency hospital admission (including potentially psychosomatic symptoms; 95% confidence intervals): derived from age group-specific Poisson regression models with separate models for each group of social strata

|  |  | **Age group** | | |
| --- | --- | --- | --- | --- |
|  |  | **11-<16** | **16-<18** | **18-<21** |
|  | *N* people | 5,628,357 | 2,769,042 | 1,710,950 |
|  | Overall | 9.5 (9.5, 9.6) | 10.6 (10.5, 10.7) | 11.2 (11.1, 11.3) |
| Gender | Male | 6.3 (6.3, 6.4) | 6.6 (6.5, 6.7) | 7.5 (7.4, 7.7) |
|  | Female | 12.9 (12.9, 13.0) | 15.0 (14.8, 15.1) | 15.4 (15.2, 15.5) |
| Region | East Midlands | 8.1 (7.9, 8.2) | 9.5 (9.3, 9.8) | 11.6 (11.2, 12.0) |
|  | East of England | 8.7 (8.6, 8.8) | 9.7 (9.5, 10.0) | 10.5 (10.1, 10.8) |
|  | London | 7.1 (7.0, 7.2) | 8.0 (7.8, 8.2) | 7.9 (7.6, 8.1) |
|  | North East | 11.6 (11.4, 11.8) | 12.6 (12.1, 13.0) | 15.1 (14.5, 15.6) |
|  | North West | 12.2 (12.1, 12.3) | 11.3 (11.1, 11.6) | 12.9 (12.6, 13.3) |
|  | South East | 8.9 (8.8, 9.0) | 11.7 (11.4, 11.9) | 11.9 (11.6, 12.2) |
|  | South West | 11.1 (11.0, 11.3) | 13.6 (13.2, 13.9) | 11.9 (11.6, 12.3) |
|  | West Midlands | 10.9 (10.7, 11.0) | 10.8 (10.5, 11.0) | 12.4 (12.0, 12.7) |
|  | Yorkshire and The Humber | 8.8 (8.7, 8.9) | 9.6 (9.4, 9.9) | 9.5 (9.2, 9.8) |
| IMD groups | 1.Most deprived | 10.5 (10.4, 10.6) | 11.6 (11.4, 11.8) | 12.8 (12.6, 13.1) |
|  | 2 | 10.0 (9.9, 10.1) | 11.1 (10.9, 11.3) | 11.8 (11.5, 12.0) |
|  | 3 | 9.7 (9.6, 9.8) | 10.8 (10.6, 11.0) | 10.9 (10.6, 11.1) |
|  | 4 | 9.0 (8.9, 9.1) | 9.8 (9.6, 10.0) | 10.3 (10.1, 10.6) |
|  | 5.Least deprived | 8.2 (8.1, 8.3) | 9.3 (9.1, 9.5) | 9.8 (9.6, 10.1) |
| FSM eligible | No | 8.8 (8.7, 8.8) | 9.7 (9.6, 9.8) | 10.4 (10.3, 10.5) |
|  | Yes | 13.4 (13.3, 13.5) | 15.1 (14.9, 15.4) | 15.3 (15.0, 15.6) |
| Racial-ethnic group (minor) | Bangladeshi | 7.1 (6.8, 7.4) | 7.3 (6.8, 7.9) | 7.1 (6.4, 7.7) |
|  | Indian | 5.9 (5.7, 6.1) | 6.3 (5.9, 6.8) | 6.5 (6.0, 7.0) |
|  | Asian (other) | 5.7 (5.5, 6.0) | 6.9 (6.3, 7.4) | 7.5 (6.8, 8.2) |
|  | Pakistani | 8.6 (8.4, 8.8) | 8.6 (8.2, 9.0) | 9.7 (9.2, 10.2) |
|  | Black African | 5.8 (5.6, 5.9) | 6.6 (6.2, 7.0) | 8.2 (7.7, 8.7) |
|  | Black Caribbean | 7.7 (7.4, 8.1) | 7.8 (7.1, 8.5) | 8.3 (7.5, 9.2) |
|  | Black (other) | 6.9 (6.5, 7.4) | 7.6 (6.6, 8.6) | 10.0 (8.7, 11.4) |
|  | Chinese | 3.7 (3.3, 4.1) | 4.9 (3.8, 5.9) | 4.6 (3.4, 5.8) |
|  | Mixed (other) | 9.0 (8.7, 9.3) | 10.0 (9.3, 10.6) | 10.5 (9.6, 11.3) |
|  | Mixed Asian & White | 9.2 (8.8, 9.6) | 10.4 (9.5, 11.2) | 10.5 (9.5, 11.5) |
|  | Mixed Black African & White | 9.3 (8.8, 9.8) | 9.8 (8.7, 10.9) | 11.1 (9.6, 12.5) |
|  | Mixed Black Caribbean & White | 11.0 (10.6, 11.4) | 12.4 (11.6, 13.2) | 11.7 (10.7, 12.6) |
|  | Other | 6.8 (6.5, 7.0) | 7.5 (6.9, 8.1) | 8.5 (7.7, 9.3) |
|  | Refused | 8.8 (8.3, 9.3) | 10.3 (9.1, 11.5) | 9.5 (8.1, 10.9) |
|  | White British | 10.4 (10.3, 10.4) | 11.5 (11.4, 11.7) | 12.1 (12.0, 12.2) |
|  | White Irish | 9.5 (8.7, 10.3) | 10.4 (8.8, 12.0) | 10.5 (8.6, 12.4) |
|  | White Irish Traveller | 12.6 (10.6, 14.7) | 10.2 (6.5, 13.9) | 18.1 (12.2, 23.9) |
|  | White (other) | 7.4 (7.2, 7.6) | 7.8 (7.4, 8.2) | 8.7 (8.3, 9.2) |
|  | White Romany Gypsy | 8.4 (7.7, 9.2) | 11.6 (9.9, 13.4) | 12.4 (10.2, 14.7) |
|  | Missing | 7.0 (5.5, 8.4) | 10.1 (6.7, 13.5) | 7.8 (4.4, 11.2) |

FSM = free school meals; IMD = index of multiple deprivation

**Table S15.** Year-adjusted incidence rate ratios of first mental health-related emergency hospital admission including potentially psychosomatic symptoms (95% confidence intervals), compared to the age- and social strata-specific mean rate: derived from age group-specific Poisson regression models with separate models for each group of social strata

|  |  | **Age group** | | |
| --- | --- | --- | --- | --- |
|  |  | **11-<16** | **16-<18** | **18-<21** |
|  | *N* people | 5,628,357 | 2,769,042 | 1,710,950 |
| Gender | Male | 0.71 (0.70, 0.71) | 0.68 (0.67, 0.68) | 0.72 (0.71, 0.72) |
|  | Female | 1.44 (1.44, 1.45) | 1.54 (1.52, 1.55) | 1.46 (1.44, 1.47) |
| Region | East Midlands | 0.86 (0.85, 0.87) | 0.91 (0.89, 0.94) | 1.05 (1.02, 1.08) |
|  | East of England | 0.92 (0.91, 0.94) | 0.93 (0.91, 0.95) | 0.95 (0.92, 0.97) |
|  | London | 0.75 (0.75, 0.76) | 0.76 (0.74, 0.78) | 0.71 (0.69, 0.73) |
|  | North East | 1.24 (1.22, 1.26) | 1.20 (1.16, 1.24) | 1.36 (1.31, 1.42) |
|  | North West | 1.30 (1.29, 1.31) | 1.08 (1.06, 1.11) | 1.17 (1.14, 1.20) |
|  | South East | 0.95 (0.94, 0.96) | 1.12 (1.10, 1.14) | 1.08 (1.05, 1.10) |
|  | South West | 1.18 (1.17, 1.20) | 1.30 (1.27, 1.33) | 1.08 (1.05, 1.11) |
|  | West Midlands | 1.16 (1.14, 1.17) | 1.03 (1.00, 1.05) | 1.12 (1.09, 1.15) |
|  | Yorkshire and The Humber | 0.94 (0.93, 0.95) | 0.92 (0.89, 0.94) | 0.86 (0.83, 0.88) |
| IMD groups | 1.Most deprived | 1.11 (1.10, 1.11) | 1.10 (1.09, 1.12) | 1.15 (1.13, 1.17) |
|  | 2 | 1.05 (1.04, 1.06) | 1.05 (1.04, 1.07) | 1.05 (1.03, 1.07) |
|  | 3 | 1.02 (1.01, 1.03) | 1.02 (1.00, 1.04) | 0.97 (0.95, 0.99) |
|  | 4 | 0.94 (0.93, 0.95) | 0.93 (0.91, 0.94) | 0.93 (0.91, 0.95) |
|  | 5.Least deprived | 0.86 (0.85, 0.87) | 0.88 (0.87, 0.90) | 0.88 (0.86, 0.90) |
| FSM eligible | No | 0.93 (0.93, 0.93) | 0.93 (0.93, 0.93) | 0.94 (0.93, 0.94) |
|  | Yes | 1.42 (1.41, 1.43) | 1.45 (1.43, 1.47) | 1.38 (1.35, 1.41) |
| Racial-ethnic group (minor) | Bangladeshi | 0.76 (0.73, 0.79) | 0.70 (0.65, 0.76) | 0.64 (0.58, 0.70) |
|  | Indian | 0.62 (0.60, 0.65) | 0.61 (0.57, 0.65) | 0.59 (0.54, 0.64) |
|  | Asian (other) | 0.61 (0.58, 0.64) | 0.66 (0.61, 0.71) | 0.68 (0.62, 0.75) |
|  | Pakistani | 0.92 (0.90, 0.94) | 0.83 (0.79, 0.87) | 0.88 (0.84, 0.93) |
|  | Black African | 0.61 (0.60, 0.63) | 0.63 (0.60, 0.67) | 0.74 (0.70, 0.79) |
|  | Black Caribbean | 0.83 (0.79, 0.86) | 0.75 (0.69, 0.82) | 0.75 (0.68, 0.83) |
|  | Black (other) | 0.74 (0.69, 0.79) | 0.73 (0.64, 0.83) | 0.91 (0.79, 1.04) |
|  | Chinese | 0.40 (0.35, 0.44) | 0.47 (0.38, 0.58) | 0.42 (0.32, 0.54) |
|  | Mixed (other) | 0.96 (0.93, 0.99) | 0.96 (0.89, 1.02) | 0.95 (0.87, 1.03) |
|  | Mixed Asian & White | 0.98 (0.94, 1.02) | 1.00 (0.92, 1.08) | 0.95 (0.86, 1.05) |
|  | Mixed Black African & White | 0.99 (0.94, 1.05) | 0.94 (0.84, 1.06) | 1.00 (0.87, 1.14) |
|  | Mixed Black Caribbean & White | 1.17 (1.13, 1.21) | 1.19 (1.12, 1.27) | 1.05 (0.97, 1.14) |
|  | Other | 0.72 (0.69, 0.75) | 0.72 (0.66, 0.78) | 0.76 (0.70, 0.84) |
|  | Refused | 0.94 (0.89, 1.00) | 0.99 (0.88, 1.11) | 0.86 (0.74, 1.00) |
|  | White British | 1.11 (1.10, 1.11) | 1.11 (1.10, 1.11) | 1.09 (1.09, 1.10) |
|  | White Irish | 1.02 (0.94, 1.10) | 1.00 (0.86, 1.16) | 0.95 (0.79, 1.13) |
|  | White Irish Traveller | 1.35 (1.15, 1.58) | 0.98 (0.68, 1.40) | 1.63 (1.18, 2.25) |
|  | White (other) | 0.79 (0.77, 0.81) | 0.75 (0.72, 0.78) | 0.79 (0.75, 0.83) |
|  | White Romany Gypsy | 0.90 (0.83, 0.98) | 1.12 (0.96, 1.30) | 1.12 (0.94, 1.34) |
|  | Missing | 0.74 (0.60, 0.91) | 0.97 (0.69, 1.36) | 0.70 (0.45, 1.09) |

FSM = free school meals; IMD = index of multiple deprivation

**Figure S13.** Year-adjusted rates of first mental health-related emergency hospital admission (including potentially psychosomatic symptoms; per 1000 person-years) with 95% confidence intervals: derived from age group-specific Poisson regression models with separate models for each group of social strata. FSM = free school meals; IMD = index of multiple deprivation

**Figure S14.** Year-adjusted incidence rate ratios (compared to the age- and social strata-specific mean rate) of first mental health-related emergency hospital admission including potentially psychosomatic symptoms with 95% confidence intervals: derived from age group-specific Poisson regression models with separate models for each group of social strata. FSM = free school meals; IMD = index of multiple deprivation
